# Associations of Distinct Dietary Indices with Global and Domain-Specific Cognition and Age-Related Heterogeneity in Older Chinese Adults

**DOI:** 10.3390/nu18203365

**Published:** 2026-10-14

**Authors:** Yiya Liu, Hua Guo

**Affiliations:** Guizhou Center for Disease Control and Prevention, Guiyang 550004, China; liuyiya163@163.com

**Keywords:** cognitive function, dietary indices, cognitive domains, older adults

## Abstract

Background: Whether distinct dietary indices are similarly associated with cognitive domains and whether these associations vary by age remain unclear. We examined the Chinese MIND diet score (cMIND), dietary diversity score, and healthy plant-based diet index (HPDI) in relation to cognition among older Chinese adults. Methods: This cross-sectional study included 9615 adults aged 65 years or older from the 2021 Chinese Longitudinal Healthy Longevity Survey. Global cognition and four cognitive domains were assessed using the Chinese Mini-Mental State Examination. Sampling-weighted linear regression models evaluated each dietary index separately. Primary associations were estimated per 1-SD increment. Diet-by-age interactions were examined for global cognition, with complementary and sensitivity analyses. Results: All three dietary indices were positively associated with global cognition. Per 1-SD increment, adjusted β coefficients were 0.355 (95% CI 0.147 to 0.563) for cMIND, 0.342 (0.119 to 0.565) for dietary diversity, and 0.388 (0.132 to 0.643) for HPDI. All three indices were positively associated with attention, executive, and visuospatial function, while dietary diversity and HPDI were additionally associated with orientation. Formal comparisons showed no evidence that standardized associations differed across indices. Significant diet-by-age interactions were observed for all three indices, with more positive associations with global cognition at older ages (all interaction *p* < 0.001). Sensitivity analyses yielded broadly consistent findings. Conclusions: Higher cMIND, dietary diversity, and HPDI scores were associated with better global cognition, with statistical evidence of age-related heterogeneity in these cross-sectional associations across all three indices. These findings highlight the importance of considering dietary dimensions and age when characterizing diet–cognition relationships in older adults.

## 1. Introduction

Population ageing has made preservation of cognitive health an increasingly important public health priority. A recent analysis based on the Global Burden of Disease Study 2021 estimated that 56.9 million people were living with Alzheimer’s disease and other dementias worldwide in 2021, about 161% more than in 1990 [1]. Cognitive decline threatens independence and quality of life, while dementia places substantial demands on families and health and social care systems. Prevention therefore remains central. The 2024 Lancet Commission estimated that 45.3% of dementia cases are associated with 14 potentially modifiable risk factors [2]. Within this broader prevention agenda, diet has attracted sustained interest because it is modifiable and is closely linked to vascular, metabolic, and inflammatory pathways relevant to brain ageing [3]. Observational studies generally associate healthier dietary patterns with better cognitive outcomes, although randomized trials have produced smaller and less consistent effects [3,4]. This has encouraged a shift from isolated nutrients toward whole dietary patterns that better reflect habitual intake. Yet dietary patterns and indices can capture fundamentally different aspects of habitual diet. The key question is therefore not only whether diet is associated with cognition, but which dietary dimensions are being captured and whether they relate to cognitive health in similar ways.

Three dietary indices illustrate this distinction. The Mediterranean–Dietary Approaches to Stop Hypertension (DASH) Intervention for Neurodegenerative Delay (MIND) diet was developed specifically for brain health by integrating elements of Mediterranean and DASH diets [5]. Observational studies have linked higher MIND adherence to slower cognitive decline and better cognitive outcomes [5], but a large randomized trial found no significant difference in cognitive change between a MIND intervention and a control diet with mild caloric restriction [6]. A recent systematic review concluded that evidence is strongest for global cognition and dementia, while findings for cognitive decline and individual cognitive domains remain mixed [7]. Population context may therefore matter. The Chinese MIND (cMIND) diet was developed to retain the brain health orientation of MIND while adapting its components to Chinese dietary characteristics [8]. Subsequent Chinese studies have reported associations with lower cognitive impairment and better cognitive performance [9,10]. Dietary diversity captures a different feature of diet. It reflects the variety of foods or food groups consumed and is widely used as an indicator of broader dietary and nutrient adequacy [11]. Its primary focus is the breadth of foods consumed, with different food groups contributing to variety irrespective of their individual nutritional quality. Cohort studies in China have linked greater dietary diversity with slower cognitive decline and lower cognitive impairment risk, including among the oldest old [12,13]. Plant-based dietary indices provide a third perspective by characterizing diets according to the types and healthfulness of plant foods consumed [14]. Prospective studies in older Chinese adults have associated healthier plant-based patterns with lower cognitive impairment risk [15,16], and a recent meta-analysis reported more favorable cognitive outcomes for healthful plant-based diets despite substantial between study heterogeneity [17]. Thus, cMIND, dietary diversity, and the adapted HPDI represent conceptually distinct dietary constructs, emphasizing brain-health-oriented dietary composition, dietary variety, and selected healthful plant-food consumption, respectively. Whether these conceptual differences translate into distinct associations with cognitive function remains uncertain.

Despite this growing evidence, three limitations remain important. First, cMIND, dietary diversity, and healthful plant-based diets have usually been studied separately. Differences in populations, dietary assessment, cognitive definitions, exposure scales, and modelling strategies make comparisons across studies difficult [8,9,12,15,16]. Moreover, although these dietary indices emphasize different aspects of diet, they share several food components, creating potential overlap in the dietary information they capture. The important question is not which index is superior, but whether distinct definitions of diet show convergent or different cognitive associations when examined in the same population under a common analytical framework. Such comparisons can help determine the extent to which conceptually distinct dietary indices provide shared or distinct information in relation to cognition. Second, many studies have focused on cognitive impairment, dementia, or a single global cognitive score [9,12,13,15,16]. Some recent studies have examined multiple cognitive domains [10,18], but this remains uncommon and has rarely been done across different dietary constructs. Global scores can conceal variation across different aspects of cognitive performance. Domain-specific analyses may therefore provide additional information on how dietary exposures relate to different cognitive functions. Third, the dose–response shape of these associations remains incompletely characterized. Linear models and broad dietary categories may miss thresholds, plateaus, or other nonlinear patterns. Recent studies of cMIND and dietary diversity have reported nonlinear or spline-based associations [13,19], but whether such patterns recur across different dietary constructs is unclear. These associations may also vary across later life. Nutritional needs, physiological reserve, multimorbidity, and vulnerability to cognitive decline change markedly across later life. Dietary associations have been demonstrated even among the oldest old, while recent cMIND analyses suggest that their magnitude may differ by age [12,19]. Whether age related heterogeneity is consistent across distinct dietary constructs, particularly into very old age, remains uncertain.

Using data from older adults in the 2021 Chinese Longitudinal Healthy Longevity Survey, we examined the cMIND diet score, dietary diversity score, and healthy plant-based diet index in relation to global and domain specific cognitive function. We estimated associations per 1-SD increment to enable parallel comparison across the three indices and further examined quartile-based and dose–response relationships. We also assessed correlations and overlap among the dietary indices and formally compared their standardized associations with cognitive outcomes. We further examined age-related heterogeneity in the associations of each dietary index with global cognitive function. By evaluating three conceptually distinct dietary constructs within the same population and analytical framework, this study aimed to clarify the extent to which their associations with cognition converge or vary across cognitive outcomes, exposure levels, and later life age.

## 2. Materials and Methods

### 2.1. Study Design and Participants

This cross-sectional study used data from the 2021 wave of the Chinese Longitudinal Healthy Longevity Survey (CLHLS). The CLHLS is an ongoing population-based longitudinal study of older adults in China that was initiated in 1998 and is coordinated by the Center for Healthy Aging and Development Studies at Peking University [20]. Follow-up surveys have subsequently been conducted in 2000, 2002, 2005, 2008 to 2009, 2011 to 2012, 2014, 2017 to 2018, and 2021. The survey collects detailed information on sociodemographic characteristics, socioeconomic conditions, lifestyle behaviors, dietary intake, physical and cognitive functioning, chronic diseases, and family and social circumstances through structured interviews. The 2021 survey covered randomly selected counties and city districts across 23 Chinese provinces and collected information from 9720 adults aged 65 years or older [21]. The CLHLS has been extensively used to study healthy ageing and longevity in Chinese older populations [20,22]. The present study used the officially released 2021 dataset obtained from the Peking University Open Research Data Platform [21]. Analyses incorporated the 2021 sampling weight supplied with the dataset.

Participants were eligible for the present analysis if they were aged 65 years or older, had complete information required to derive the three dietary indices, had valid cognitive outcome data, and had a valid positive 2021 sampling weight. Of the 9720 participants in the 2021 CLHLS, 101 were excluded because of incomplete dietary information, and a further 4 were excluded because of missing or invalid sampling weights, leaving 9615 participants in the final analytic sample. Participants with missing covariate data were retained because these values were subsequently handled using multiple imputation. The CLHLS was approved by the Biomedical Ethics Committee of Peking University (IRB00001052-13074) and was conducted in accordance with the ethical principles of the Declaration of Helsinki, and written informed consent was obtained from all participants or their legal representatives. The present study involved secondary analysis of the publicly released and de-identified 2021 CLHLS data.

### 2.2. Dietary Assessment and Dietary Indices

Dietary information was obtained from food-frequency questions included in the 2021 CLHLS questionnaire. Participants reported their habitual consumption of a range of foods commonly consumed in the Chinese diet, including vegetables, fruits, staple foods, meat, fish and seafood, eggs, legumes, dairy products, nuts, tea, garlic, sugar-containing foods, and other dietary items. Using these data, we constructed three dietary indices representing distinct dietary constructs. These were the cMIND diet score, the dietary diversity score, and the healthy plant-based diet index. The three indices were calculated independently rather than entered as components of a common score. Detailed definitions of the dietary components and the scoring procedures used for each index are provided in Supplementary Table S1.

The cMIND diet score was constructed with reference to the cMIND dietary pattern developed for older Chinese adults by Huang et al. [8]. The cMIND pattern was adapted from the original Mediterranean Dietary Approaches to Stop Hypertension Intervention for Neurodegenerative Delay diet to better reflect foods and dietary habits relevant to the Chinese population. The scoring system emphasizes dietary components considered favorable for cognitive health and assigns scores in the opposite direction for unfavorable components [8]. Corresponding dietary items available in the 2021 CLHLS were mapped to the cMIND components according to the established scoring framework. Component scores were summed to generate the overall cMIND score. Higher scores represented greater adherence to the cMIND dietary pattern.

Dietary diversity was assessed using an approach adapted from the CLHLS-based study by Ren et al. [23]. Dietary diversity was defined according to daily consumption of 12 food categories, including fruits, vegetables, grains, meat, fish and seafood, eggs, dairy products, legumes, sweets, garlic, nuts, and tea. One point was assigned for daily consumption of each food category and zero points otherwise. The points were summed to generate a dietary diversity score ranging from 0 to 12, with higher scores indicating consumption of a broader range of food categories. The score was therefore intended primarily to capture food-group variety rather than the nutritional quality of individual foods. In the original study by Ren et al., consumption of at least five of the 12 food categories daily was additionally used to define an ideal diet for construction of a healthy lifestyle score [23]. In the present study, however, the full continuous dietary diversity score was retained to preserve information across its distribution.

The healthy plant-based diet index (HPDI) was constructed with reference to the plant-based dietary assessment used by Zou et al. [24] and was adapted to the dietary items available in the 2021 CLHLS. Five healthful plant-food components available in the CLHLS, including fresh fruit, fresh vegetables, bean products, garlic, and tea, were included. For each component, consumption reported as rarely or never, sometimes or occasionally, and always or almost every day was assigned 0, 1, and 2 points, respectively. Component scores were summed to obtain an HPDI ranging from 0 to 10, with higher scores indicating more frequent consumption of the included healthful plant foods. This adapted score was designed to characterize the frequency and overall level of consumption of these selected healthful plant foods. Detailed scoring criteria are provided in Supplementary Table S1.

### 2.3. Assessment of Cognitive Function

Cognitive function was assessed using the Chinese version of the Mini-Mental State Examination (C-MMSE), which has been used extensively in the CLHLS [22,23]. The CLHLS cognitive assessment contains 24 questions with a maximum global score of 30 points. For each C-MMSE item, responses were coded as correct, incorrect, or “unable to answer” according to the CLHLS questionnaire coding scheme. Correct responses received the corresponding points, whereas incorrect responses and responses coded as “unable to answer” received zero points for the corresponding item. Selected tasks allow more than one point. Higher scores indicate better cognitive performance. Previous CLHLS studies have supported the use of C-MMSE items to characterize domain-specific cognitive performance. Factor-analytic studies have identified multidimensional structures encompassing orientation, memory, attention/calculation, and language, and previous CLHLS analyses have similarly grouped registration and recall into a memory domain [25,26,27]. Consistent with this evidence, cognitive performance in the present study was examined both globally and across four domains. Orientation was derived from items assessing orientation to time and place and ranged from 0 to 5. Memory incorporated immediate registration and delayed recall and ranged from 0 to 6. Attention, executive, and visuospatial function incorporated tasks assessing attention and calculation, verbal fluency, and visuospatial ability and ranged from 0 to 13. Language was derived from language-related items and ranged from 0 to 6. Global cognitive function was calculated as the sum of these four domain scores and ranged from 0 to 30. All cognitive scores were analyzed as continuous outcomes, with higher values representing better cognitive function.

### 2.4. Covariates

Covariates were selected a priori on the basis of previous CLHLS studies and their plausible relationships with both dietary behaviors and cognitive function [8,22,23]. Sociodemographic variables included age group, sex, residence, marital status, and education. Age was categorized as 65 to 74, 75 to 84, 85 to 99, and 100 years or older. Sex was classified as male or female, residence as rural or urban, and marital status as married and living with a spouse or other. Education was categorized as no formal education, 1 to 6 years, or more than 6 years.

Health-related factors included number of chronic diseases and disability in activities of daily living (ADL). The chronic disease count was based on 13 self-reported physician-diagnosed conditions, including hypertension, diabetes, heart disease, stroke, chronic respiratory disease, tuberculosis, cataract, glaucoma, cancer, gastric ulcer, Parkinson’s disease, bedsores, and arthritis. Participants were classified as having no chronic disease, one chronic disease, or two or more chronic diseases. In the fully adjusted primary model, five individual chronic conditions—hypertension, diabetes, heart disease, stroke/cerebrovascular disease, and Parkinson’s disease—were included separately instead of the chronic disease count. Each condition was classified as present or absent. ADL disability was assessed using six basic activities comprising bathing, dressing, toileting, indoor mobility or transferring, bowel or bladder control, and eating. Participants who required assistance with at least one of these activities were classified as having ADL disability [23].

Socioeconomic covariates included pension or retirement income and medical insurance. Self-rated economic status was additionally included and categorized as rich or very rich, average, or poor or very poor. Lifestyle covariates included current smoking and current drinking. Current regular exercise and social participation were also included and classified as yes or no. Depressive symptoms were assessed using the nine-item version of the Center for Epidemiologic Studies Depression Scale (CESD-9), as previously used in studies based on CLHLS data [28]. Items were coded in the direction of greater symptom severity, with positively worded items reverse-coded before summation; thus, higher total scores indicated more severe depressive symptoms. The total CESD-9 score was included as a continuous covariate. Body mass index (BMI) was calculated as weight in kilograms divided by height in meters squared and was included as a continuous covariate. Binary covariates were coded as indicator variables where applicable, while categorical variables with more than two levels were represented using the categories specified above.

### 2.5. Statistical Analysis

Descriptive analyses were conducted using the original data before multiple imputation. Continuous variables are presented as means and standard deviations (SDs), and categorical variables as numbers and percentages. Percentages were calculated among participants with non-missing data for the corresponding variable. Descriptive statistics were not weighted and were used to characterize the analytic sample. Missing values in the three dietary indices, cognitive outcomes, and sampling weight were not imputed. Missing covariate values were handled using multiple imputation by chained equations (MICE) [29]. Variable-specific missingness is reported in Supplementary Table S2. Twenty imputed datasets were generated, with 30 iterations for each imputation procedure. Incomplete CESD-9 and BMI values were imputed using predictive mean matching. Binary incomplete variables were imputed using logistic regression and nominal multicategorical variables using multinomial logistic regression, as appropriate. Variables without missing values were allowed to contribute as predictors of incomplete covariates. Participant identifiers were excluded from the predictor matrix, and global cognitive function was not used as a predictor because it was deterministically derived from the four cognitive domain scores. Convergence was assessed using trace plots, potential scale reduction factors, and chain autocorrelation diagnostics. Estimates from analyses of the 20 completed datasets were combined according to Rubin’s rules [30]. The 2021 sampling weight was applied to all regression analyses.

Sampling-weighted analyses were implemented using the survey framework in R [31]. Weighted linear regression models were fitted separately for each dietary index and each cognitive outcome. Thus, cMIND, dietary diversity, and HPDI were evaluated in separate models in the primary analyses and were not mutually adjusted for one another. Four sequential adjustment models were fitted in the full analytical sample (N = 9615). Model 1 was unadjusted. Model 2 adjusted for age group, sex, residence, marital status, education, pension or retirement income, medical insurance, and self-rated economic status. Model 3 additionally adjusted for number of chronic diseases (0, 1, or ≥2), ADL disability, current smoking, current drinking, regular exercise, social participation, depressive symptom score (CESD-9), and BMI. Model 4 replaced the number of chronic diseases with hypertension, diabetes, heart disease, stroke/cerebrovascular disease, and Parkinson’s disease, while retaining the other Model 3 covariates. Model 4 was designated as the fully adjusted primary model. The primary analyses expressed the association with cognition per one sampling-weighted SD increment in each dietary index. This standardized parameterization was selected to facilitate parallel assessment of the three indices despite their different scoring ranges. The sampling-weighted SDs were 1.580 for the cMIND score, 2.031 for the dietary diversity score, and 1.833 for the HPDI. Results are reported as regression coefficients (β) with 95% confidence intervals (CIs), with a positive coefficient indicating a higher cognitive score associated with a higher dietary index.

Sampling-weighted and unweighted Pearson correlations were calculated to assess correlations among the three dietary indices. In an additional mutually adjusted analysis, all three dietary indices were centered at their sampling-weighted means and scaled by their sampling-weighted SDs, then entered simultaneously with the Model 4 covariates. Collinearity was assessed using variance inflation factors across the imputed datasets. Pairwise contrasts and a global Wald test formally assessed equality of the standardized dietary coefficients within these models, accounting for their covariance. Contrast estimates and variances were combined using Rubin’s rules, and the global comparison used a pooled multivariate Wald test.

Complementary analyses evaluated each dietary index per one-point increment and according to sampling-weighted quartiles. These analyses used Model 4 adjustment. Weighted quartile cut points were derived from the observed distribution of each index. Participants in the lowest quartile served as the reference group, and adjusted differences in cognitive scores were estimated for the second, third, and fourth quartiles. Tests for linear trend were conducted by assigning each participant the sampling-weighted median value of the quartile to which they belonged and modeling this value as a continuous variable. This procedure allowed the trend test to reflect the observed spacing of dietary scores across quartiles rather than assuming equal distances between ordinal quartile categories.

Restricted cubic spline models were used to characterize potential nonlinear dose–response associations [32]. For each dietary index, four knots were positioned at its sampling-weighted 5th, 35th, 65th, and 95th percentiles. The sampling-weighted median value of the corresponding dietary index was used as the reference. The spline models used Model 4 covariate adjustment. Wald tests were performed to assess the overall association and evidence of nonlinearity. For graphical presentation, predicted associations and 95% CIs were plotted over the sampling-weighted 1st to 99th percentile range of each dietary index to reduce instability caused by sparse observations at the extreme tails of the distributions. Exposure SDs, quartile cut points and medians, and spline knots and references were calculated once from the original analytical sample and held fixed across imputed datasets.

Age-related heterogeneity was examined for global cognitive function. Each dietary index was modeled separately together with its interaction with continuous age. Age was centered at 80 years to improve interpretation of the main dietary association. Because continuous age was explicitly included in these interaction models, categorical age group was excluded, while all remaining Model 4 covariates were retained. The statistical significance of the interaction term was used to assess evidence of age-related heterogeneity. For visualization, adjusted associations between each dietary index and global cognitive function were estimated at ages 70, 80, 90, and 100 years.

Sensitivity analyses included three components. First, estimates from Models 3 and 4 were compared to assess the influence of chronic-disease-count versus disease-specific adjustment. Second, Model 4 was repeated among participants with complete data for all Model 4 covariates, without imputation and using the original sampling-weighted SDs. Third, Model 5 excluded participants whose MMSE scores were at or below the weighted 5th percentile within their 5-year age group and/or who had reported dementia in the original data, leaving 8268 participants. Age-specific thresholds were calculated once from the original data, and missing dementia status was not treated as reported dementia. The same participants were excluded from all 20 completed datasets without reimputation. In this sample, the primary linear analyses, restricted cubic spline analyses, and diet-by-age interaction analyses were repeated using the corresponding Model 4 adjustment, with continuous age replacing age group in the interaction models. The original exposure SDs, spline knots, and reference values were retained.

All statistical tests were two-sided, and *p* < 0.05 was considered statistically significant. Analyses were conducted in R version 4.5.2 using the mice, survey, mitools, Hmisc, and related packages.

## 3. Results

### 3.1. Participant Characteristics

A total of 9615 adults aged 65 years or older were included in the analysis (Table 1). The mean age was 84.62 years (SD, 10.80), with 21.0% of participants aged 65 to 74 years, 31.2% aged 75 to 84 years, 36.0% aged 85 to 99 years, and 11.8% aged 100 years or older. Women accounted for 53.7% of the analytic sample, and 69.4% of participants lived in urban areas. Overall, 42.2% were married and living with a spouse, 38.9% had two or more chronic diseases, and 28.5% had ADL disability. In addition, 40.3% reported current regular exercise and 14.8% reported social participation. The mean BMI was 23.24 kg/m^2^ (SD, 6.37), and the mean CESD-9 score was 10.04 (SD, 5.81). The mean cMIND score was 4.23 (SD, 1.62), the mean dietary diversity score was 4.16 (SD, 2.03), and the mean HPDI was 5.31 (SD, 1.96). The mean global cognitive score was 21.57 (SD, 10.17). Mean scores for orientation, memory, attention, executive and visuospatial function, and language were 3.86 (SD, 1.86), 4.25 (SD, 2.37), 8.73 (SD, 4.49), and 4.73 (SD, 2.18), respectively. Among the five chronic conditions included individually in the fully adjusted model, hypertension was reported by 42.3% of participants, diabetes by 10.5%, heart disease by 17.2%, stroke/cerebrovascular disease by 11.2%, and Parkinson’s disease by 1.4%. Reported dementia was present in 4.4% of participants. Missingness varied across covariates and was highest for CESD-9 (17.4%) and BMI (5.8%); variable-specific missingness is reported in Supplementary Table S2.

### 3.2. Associations of Dietary Indices with Cognitive Function

In fully adjusted sampling-weighted models, all three dietary indices were positively associated with global cognitive function (Table 2). For each 1-SD higher cMIND score, the global cognitive score was 0.355 points higher (β = 0.355, 95% CI 0.147 to 0.563). Corresponding estimates were 0.342 points for dietary diversity (β = 0.342, 95% CI 0.119 to 0.565) and 0.388 points for HPDI (β = 0.388, 95% CI 0.132 to 0.643). For domain-specific cognition, a 1-SD higher cMIND score was positively associated with attention, executive and visuospatial function (β = 0.238, 95% CI 0.126 to 0.349). Associations of cMIND with orientation (β = 0.036, 95% CI −0.000 to 0.072), memory (β = 0.047, 95% CI −0.008 to 0.102), and language (β = 0.034, 95% CI −0.006 to 0.075) were not statistically significant. Dietary diversity was also positively associated with orientation (β = 0.045, 95% CI 0.010 to 0.080), attention, executive and visuospatial function (β = 0.252, 95% CI 0.123 to 0.381). Its associations with memory (β = 0.020, 95% CI −0.042 to 0.081) and language (β = 0.025, 95% CI −0.020 to 0.071) were not statistically significant. HPDI was positively associated with orientation (β = 0.055, 95% CI 0.009 to 0.101), and attention, executive and visuospatial function (β = 0.237, 95% CI 0.102 to 0.371). The associations of HPDI with memory (β = 0.065, 95% CI −0.002 to 0.133) and language (β = 0.031, 95% CI −0.021 to 0.083) were not statistically significant.

Results based on 1-point increments were consistent with the standardized analyses, with all three dietary indices positively associated with global cognitive function and attention, executive, and visuospatial function. Dietary diversity and HPDI were additionally associated with orientation, whereas associations with memory and language were generally not statistically significant (Supplementary Tables S3–S5).

Quartile analyses were broadly consistent with the continuous analyses, with higher levels of all three dietary indices generally associated with better global cognition and attention, executive, and visuospatial function (Supplementary Table S6). Associations with orientation were observed for dietary diversity and HPDI, whereas evidence for memory and language was less consistent across indices. The three dietary indices were moderately to strongly correlated (sampling-weighted r = 0.685–0.729; Supplementary Table S7 and Figure S1). When all three standardized indices were mutually adjusted, none showed an independent association with cognitive outcomes, while collinearity remained moderate (VIFs, 2.4–2.7; Supplementary Table S8). Pairwise contrasts and global Wald tests showed no evidence that the standardized associations differed among the three dietary indices for any cognitive outcome (all global *p* ≥ 0.166; Supplementary Table S9).

### 3.3. Dose–Response Associations

**Figure 1:**
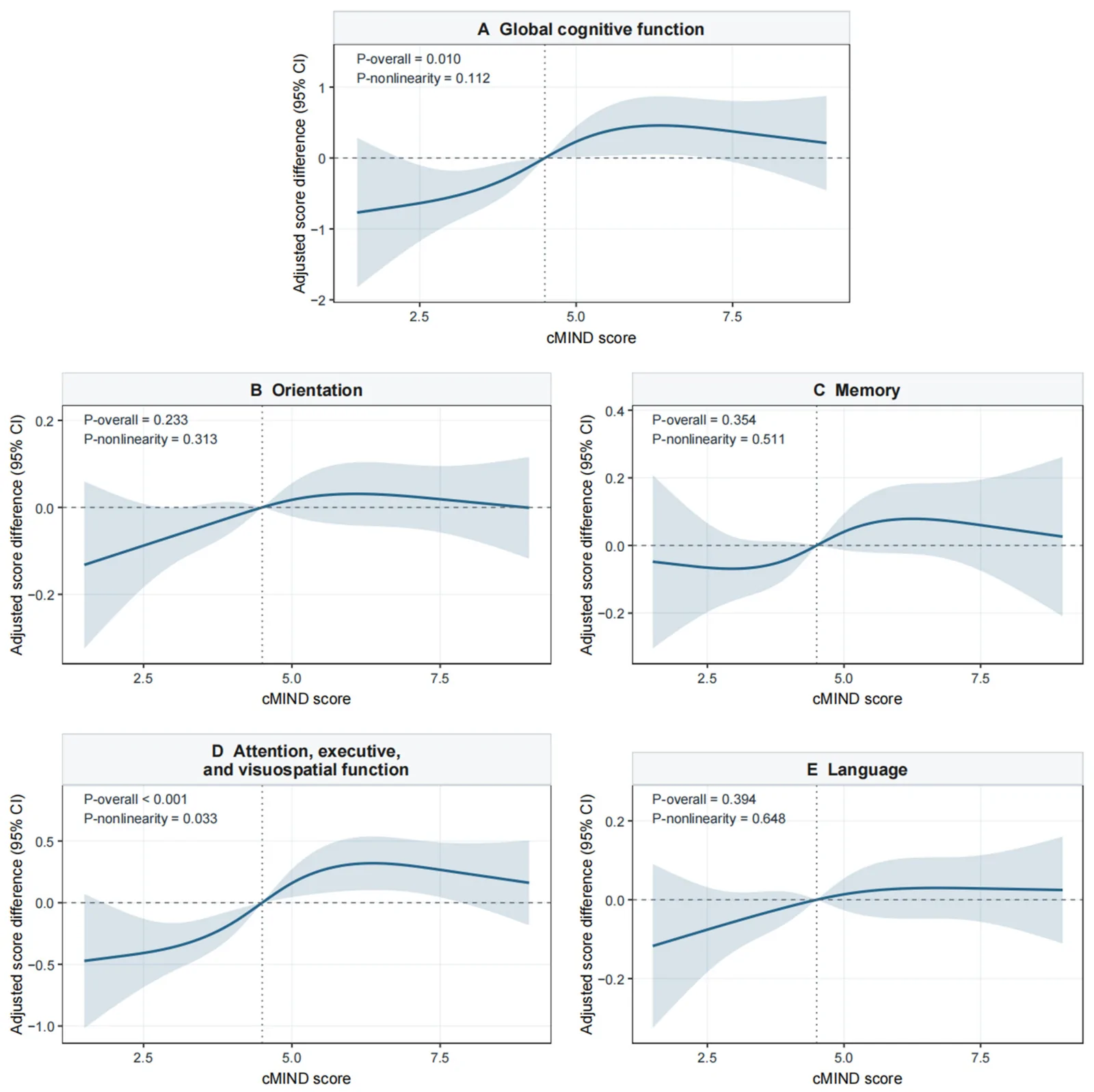
Dose–response associations of cMIND score with global and domain-specific cognitive function. Restricted cubic spline associations of Chinese MIND diet (cMIND) score with global cognitive function (**A**), orientation (**B**), memory (**C**), attention, executive, and visuospatial function (**D**), and language (**E**). Solid lines indicate adjusted estimates and shaded areas indicate 95% confidence intervals. The vertical dotted line indicates the weighted median cMIND score used as the reference, and the horizontal dashed line indicates no difference in cognitive score. P-overall tests the overall association and P-nonlinear tests departure from linearity. Models were weighted and fully adjusted using Model 4 covariates.

**Figure 2:**
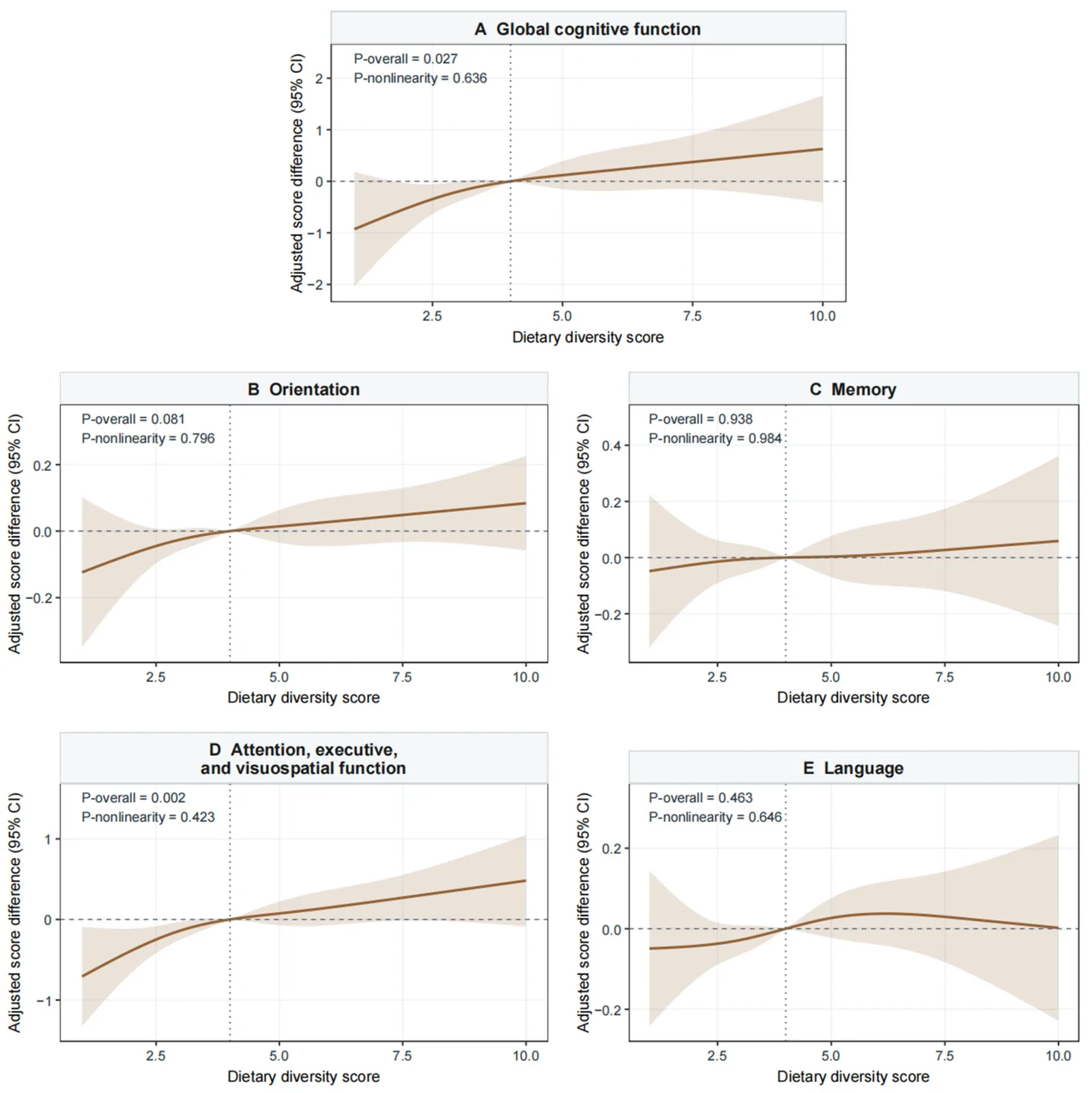
Dose–response associations of dietary diversity with global and domain-specific cognitive function. Restricted cubic spline associations of dietary diversity score with global cognitive function (**A**), orientation (**B**), memory (**C**), attention, executive, and visuospatial function (**D**), and language (**E**). Solid lines indicate adjusted estimates and shaded areas indicate 95% confidence intervals. The vertical dotted line indicates the weighted median dietary diversity score used as the reference, and the horizontal dashed line indicates no difference in cognitive score. P-overall tests the overall association and P-nonlinear tests departure from linearity. Models were weighted and fully adjusted using Model 4 covariates.

**Figure 3:**
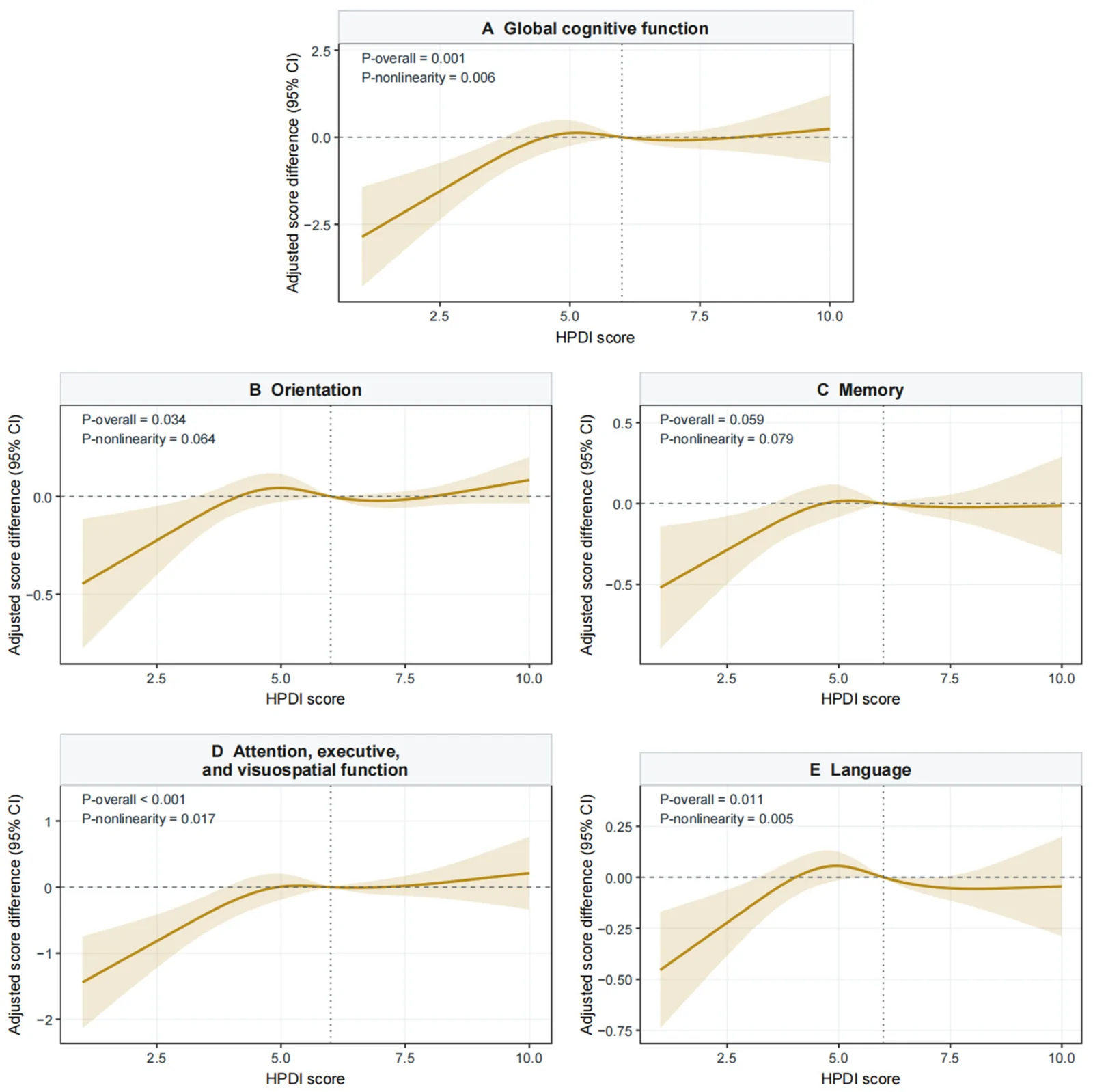
Dose–response associations of the healthy plant-based diet index with global and domain-specific cognitive function. Restricted cubic spline associations of the healthy plant-based diet index (HPDI) with global cognitive function (**A**), orientation (**B**), memory (**C**), attention, executive, and visuospatial function (**D**), and language (**E**). Solid lines indicate adjusted estimates and shaded areas indicate 95% confidence intervals. The vertical dotted line indicates the weighted median HPDI used as the reference, and the horizontal dashed line indicates no difference in cognitive score. P-overall tests the overall association and P-nonlinear tests departure from linearity. Models were weighted and fully adjusted using Model 4 covariates.

Restricted cubic spline analyses showed positive dose–response associations for several cognitive outcomes, with evidence that the shape of these associations varied across dietary indices and domains (Figure 1, Figure 2 and Figure 3 and Supplementary Table S10). For cMIND, significant overall associations were observed with global cognition and attention, executive and visuospatial function. Evidence of nonlinearity was observed for attention, executive and visuospatial function (P-nonlinearity = 0.033), whereas the association with global cognition showed no significant departure from linearity (Figure 1). For dietary diversity, significant overall associations were observed for global cognition and attention, executive and visuospatial function. No evidence of nonlinearity was observed for any of the five cognitive outcomes (Figure 2). HPDI showed significant overall associations with global cognition and orientation, attention, executive and visuospatial function, and language. Significant departures from linearity were observed for global cognition, attention, executive and visuospatial function, and language, whereas no statistically significant nonlinearity was detected for orientation or memory (Figure 3). Overall, the spline analyses supported associations of all three dietary indices with global cognition and attention, executive and visuospatial function, with evidence of nonlinearity for selected outcomes of cMIND and HPDI but not dietary diversity.

### 3.4. Age-Related Heterogeneity

**Figure 4:**
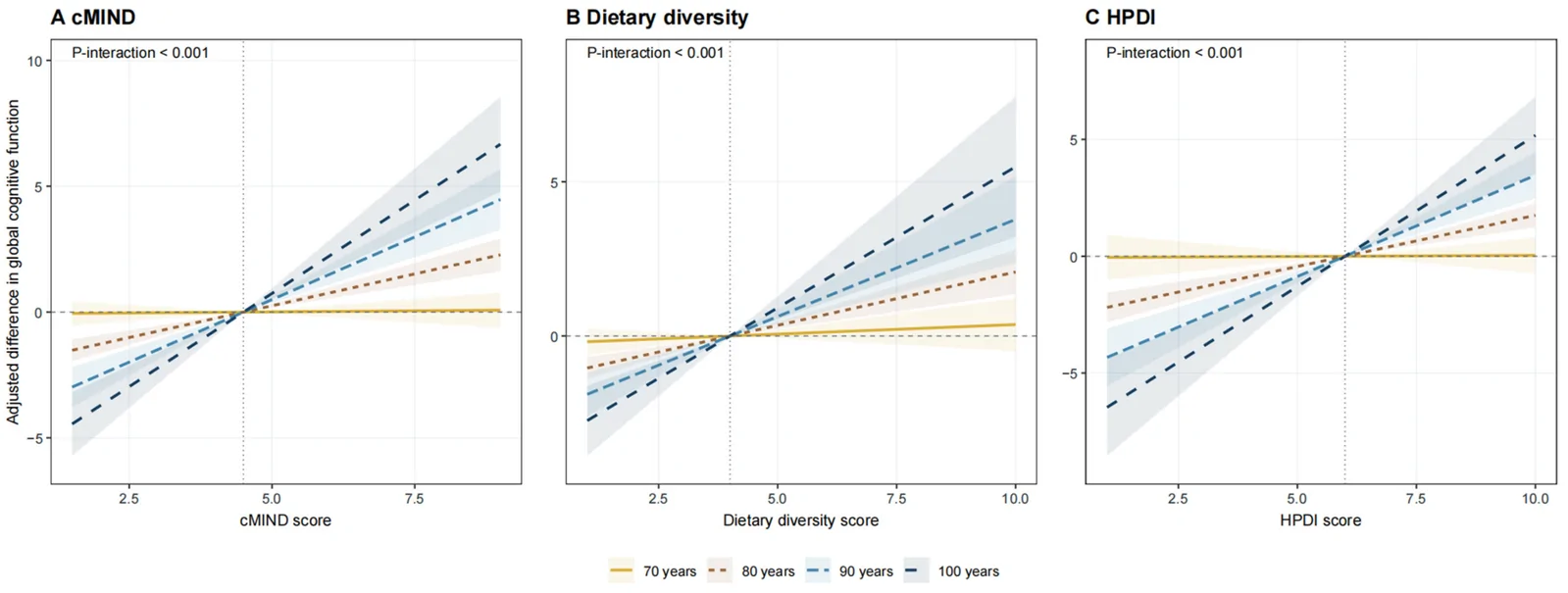
Age-related heterogeneity in associations between dietary indices and global cognitive function. Adjusted associations of cMIND score (**A**), dietary diversity score (**B**), and HPDI (**C**) with global cognitive function at ages 70, 80, 90, and 100 years. Lines indicate estimated associations and shaded areas indicate 95% confidence intervals. Horizontal dashed lines indicate the null value (difference = 0), and vertical dotted lines indicate the reference value of each dietary index used for estimating adjusted score differences. Models used continuous age and diet-by-age interaction terms with the remaining Model 4 covariates. *p* values are for diet-by-age interaction.

Significant interactions with age were observed for all three dietary indices in relation to global cognitive function (Table 3). The interaction coefficient was 0.049 per year for cMIND (95% CI 0.032 to 0.065), 0.028 per year for dietary diversity (95% CI 0.013 to 0.044), and 0.043 per year for HPDI (95% CI 0.025 to 0.061), with all interaction tests yielding *p* < 0.001. These positive interaction coefficients indicated that the positive association between each dietary index and global cognition was stronger at older ages. Model-based estimates illustrated this pattern at ages 70, 80, 90, and 100 years (Figure 4). Across all three indices, the estimated associations became progressively more positive with increasing age, indicating age-related heterogeneity in their cross-sectional associations with global cognitive function.

### 3.5. Sensitivity Analyses

Estimates were similar when chronic diseases were adjusted for using the disease count (Model 3) or five individual chronic conditions (Model 4) (Supplementary Table S11). Complete-case analyses (N = 7289) were also broadly consistent with the multiply imputed analyses (Supplementary Table S12). After excluding participants with age-specific very low MMSE scores and/or reported dementia (N = 8268; Supplementary Table S13), all three dietary indices remained positively associated with global cognitive function and attention, executive, and visuospatial function (Table 4). Associations with memory remained nonsignificant for all three indices, whereas associations with several other domains varied across indices. The corresponding spline analyses yielded broadly similar dose–response patterns (Supplementary Figures S2–S4), and diet-by-age interactions remained significant for cMIND, dietary diversity, and HPDI (all *p* < 0.001; Supplementary Table S14 and Figure S5).

## 4. Discussion

In this study of 9615 Chinese adults aged 65 years or older, we evaluated three dietary constructs within the same population and analytical framework and related them to global and domain-specific cognitive function. cMIND, dietary diversity, and HPDI were all positively associated with global cognition. All three indices were also positively associated with attention, executive, and visuospatial function, while dietary diversity and HPDI were additionally associated with orientation. The standardized associations showed substantial overlap across the three indices and cognitive outcomes. The dose–response analyses supported associations with several cognitive outcomes, with evidence of nonlinearity for selected outcomes of cMIND and HPDI but not dietary diversity. Significant statistical interactions with age were observed for all three indices, with more positive associations with global cognition at older ages. Together, these findings suggest that conceptually distinct dietary indices capture overlapping diet–cognition associations while also highlighting variation across cognitive outcomes, dose–response forms, and later-life age.

The positive associations of all three dietary indices with global cognition are consistent with broader evidence linking dietary patterns with cognitive health. Previous studies have reported favorable cognitive associations for cMIND, dietary diversity, and plant-based dietary patterns separately [8,9,10,11,12,13,15,16,17,18,19,33]. A recent longitudinal analysis that examined several dietary indices in the same older Chinese population also found that HPDI, dietary diversity, and MIND scores were each associated with a lower probability of transition from normal cognition to mild cognitive impairment [34]. Our findings extend this evidence by evaluating these dietary constructs on a common standardized scale and directly comparing their associations within the same population. cMIND evaluates adherence to a composition selected specifically for cognitive health, dietary diversity captures the breadth of daily food-group intake, and the HPDI used here captures the frequency of selected healthful plant foods. Despite these conceptual differences, the three indices were moderately to strongly correlated in the present study. Their standardized associations with cognitive outcomes were broadly comparable, and the associations were attenuated when all three indices were entered simultaneously. These findings suggest that conceptually distinct dietary indices may capture substantial shared dietary information relevant to cognitive performance while retaining different meanings in how habitual diet is characterized.

The domain-specific results provide a complementary perspective to the global cognitive findings. In the fully adjusted models, all three dietary indices were positively associated with attention, executive, and visuospatial function, while associations with other domains were less consistent. Importantly, the observed variation across index–domain associations did not translate into clear differences among the three dietary indices. Thus, variation in statistical significance across individual index–domain associations should not be interpreted as evidence that one dietary index has a stronger domain-specific association than another. Studies of other healthy dietary patterns likewise show that global cognition and individual domains do not necessarily move together. Greater adherence to an EAT-Lancet dietary pattern was associated more consistently with executive function, attention, and working memory than with episodic memory [35], and longitudinal dietary-quality trajectories have also shown different associations across memory and other cognitive domains [36]. The present findings therefore support examining domain-specific cognition alongside global cognition while interpreting differences across domains cautiously. The magnitudes of the observed associations were modest: even for global cognition, a 1-SD higher dietary index corresponded to differences of approximately 0.34–0.39 points on the 30-point cognitive scale. These estimates characterize cross-sectional population differences and should be interpreted in relation to their magnitude as well as their statistical significance. These findings also have a methodological implication. Global cognitive scores are useful summary outcomes, but they can conceal variation in associations across different aspects of cognitive performance. Future nutritional epidemiology studies should therefore consider both global and domain-specific cognitive outcomes when characterizing diet–cognition relationships.

Age-related heterogeneity was another notable finding of this study. Statistically significant diet-by-age interactions were observed for cMIND, dietary diversity, and HPDI, and the direction was consistent across all three indices. The association with global cognition was weakest at younger old ages and became progressively more positive at older ages. This consistency across conceptually distinct dietary indices suggests that the observed statistical interaction was not specific to a single dietary construct. Existing evidence has not established a single age pattern for diet and cognition. Cai et al. found a stronger longitudinal association of cMIND with incident cognitive impairment among younger older adults [19], whereas age did not significantly modify cognitive response in a recent analysis of the MIND intervention trial [37]. Our study addresses a different question. We examined variation in the cross-sectional association between diet and the level of cognitive performance across a continuous age range extending to centenarians. The contrasting findings across studies suggest that age-related heterogeneity may depend on the dietary exposure, cognitive outcome, study design, and age distribution of the population examined.

There are several reasons why this age gradient deserves attention. At advanced ages, dietary intake may increasingly reflect the ability to maintain adequate and varied food consumption in the presence of functional decline, frailty, multimorbidity, and nutritional vulnerability. Previous longitudinal evidence has shown that plant-based diet and frailty jointly characterize subsequent cognitive decline [38], while poor nutritional status is associated with adverse cognitive outcomes in older populations [39]. Changes in functional independence, food access, meal preparation, and dietary habits across later life may therefore contribute to the observed age-related pattern. At the younger end of old age, measurement may also play a role. The MMSE has recognized limitations in distinguishing relatively subtle differences among people with relatively preserved cognition, which may influence comparisons across age groups [40]. These factors provide a plausible context for the more positive associations observed at older ages, although they do not fully explain them. Selective survival may also contribute, because individuals surviving to very old ages represent a selected population with distinctive health, functional, and lifestyle characteristics. Taken together, these considerations suggest that age-related heterogeneity warrants explicit evaluation in diet–cognition research, particularly in cohorts spanning a wide range of later life. Prospective studies with repeated assessments of diet, cognition, nutritional status, and functional health are needed to clarify the processes underlying these statistical interactions.

The dose–response findings provide a complementary message. There was no common exposure-response shape across the three indices. cMIND was largely compatible with linear associations, apart from attention, executive and visuospatial function. Dietary diversity showed no evidence of nonlinearity for any cognitive outcome. For this score, the data therefore were compatible with relatively graded associations across the observed range. HPDI showed a different pattern, with significant nonlinearity for global cognition, attention, executive and visuospatial function, and language. Recent dose–response evidence for plant-based dietary indices has similarly shown that their associations with cognitive impairment and dementia may be nonlinear [41]. These findings illustrate the value of examining the functional form of diet-cognition associations rather than relying solely on linear terms or predefined categories. For cMIND and dietary diversity, retaining the full score preserves information across much of the distribution. For HPDI, the estimated cognitive difference associated with an additional score point varies across the distribution, but the current analysis does not identify a single optimal score. The broader point is that dose–response form may differ across dietary constructs and cognitive outcomes and is therefore informative when characterizing these associations.

These results have several implications for dietary assessment and future research. First, dietary diversity is an indicator of dietary breadth whereas cMIND provides information on adherence to a cognitive-health-oriented dietary pattern, and the adapted HPDI characterizes the frequency of selected healthful plant-food consumption. These conceptual distinctions remain relevant to the interpretation of dietary scores even though their associations with cognition overlapped substantially in the present analyses. Current geriatric nutrition guidance emphasizes maintaining adequate intake, preventing malnutrition, and tailoring dietary management to the circumstances of older adults [42]. Future studies may therefore benefit from clearly distinguishing the dietary dimensions represented by different indices while evaluating the extent of information they share.

Second, age and cognitive domain may be important considerations in the design of future diet–cognition research. The variation observed across global and domain-specific cognitive outcomes highlights the value of assessing cognition beyond a single summary measure. Trials and prospective cohorts could therefore incorporate sufficiently sensitive domain-specific outcomes alongside global cognitive measures. Likewise, the statistical interactions observed across later life support examining a broad age range, including the oldest old, in future prospective studies. The broader intervention literature also places diet within a multidomain prevention framework. Structured interventions combining dietary improvement with physical activity, cognitive training, and vascular risk management have improved cognitive outcomes in older adults [43,44]. Long-term observational evidence further links healthy dietary and lifestyle patterns with healthier ageing and cognitive outcomes [45,46,47]. At the same time, recent harmonized analyses across multiple cohorts show that diet-cognition associations can be modest and vary by cognitive outcome and dietary pattern [48]. Together, these findings support further prospective and intervention research that distinguishes dietary constructs, incorporates domain-specific cognitive assessment, and examines age-related heterogeneity.

This study has several strengths. The three dietary indices were evaluated in the same population using the same cognitive assessment, covariate adjustment, sampling weights, and statistical framework, allowing their associations to be compared on a common basis. Standardization to sampling-weighted SDs enabled direct comparison across indices with different scoring ranges, while formal coefficient comparisons, correlation analyses, and mutually adjusted models provided complementary assessments of their shared and distinct information. Cognition was analyzed both globally and across four domains, allowing associations across different aspects of cognitive performance to be characterized. Associations were also tested using standardized increments, original score units, weighted quartiles, and restricted cubic splines, allowing consistency and dose–response form to be evaluated. Multiple imputation retained participants with incomplete covariate data, and the robustness of the findings was examined using alternative chronic-disease adjustment, complete-case analysis, and exclusion of participants with age-specific very low MMSE scores or reported dementia. The wide age distribution of the CLHLS, including large numbers of adults aged 80 years and older, further enabled continuous assessment of age-related heterogeneity across a range rarely represented in diet-cognition studies.

Several limitations should be acknowledged. First, the cross-sectional design does not establish temporal direction, and poorer cognition may itself lead to changes in food choice, meal preparation, and dietary variety. This possibility may be particularly relevant among very old adults with functional limitations. Although the main findings were broadly maintained after excluding participants with age-specific very low MMSE scores or reported dementia, reverse causation remains possible. Second, dietary assessment was based on relatively simple food-frequency items without detailed information on portion size, energy intake, or nutrient intake. The simplified five-component HPDI should therefore be interpreted as the specific plant-food measure used in this study rather than as a conventional comprehensive HPDI. The dietary indices consequently characterize patterns and frequencies of food consumption more directly than quantitative nutrient or energy intake. Third, cognitive domains were derived from the C-MMSE rather than a full neuropsychological battery, which limits the precision with which individual cognitive abilities were measured. Although previous factor-analytic studies using CLHLS data support multidimensional interpretation of C-MMSE items, the domain scores used here remain screening-based measures rather than comprehensive domain-specific neuropsychological assessments. Fourth, the expanded models accounted for physical activity, depressive symptoms, BMI, social participation, socioeconomic factors, functional status, and major chronic diseases; nevertheless, residual confounding remains possible, particularly from medication use, frailty and nutritional status, and other incompletely measured health and social factors. Fifth, the large number of very old participants makes this cohort particularly valuable for studying late life, but selective survival should be considered when interpreting age-related heterogeneity. The three dietary indices also shared several food components and were moderately to strongly correlated. Although correlations, collinearity diagnostics, mutually adjusted models, and formal coefficient comparisons were used to characterize this overlap, the indices should still be interpreted as related representations of habitual diet rather than fully independent exposures. Finally, generalizability requires consideration because the CLHLS population consists of Chinese older adults and includes substantial representation of the oldest old. Dietary habits, educational experiences, socioeconomic conditions, healthcare access, and patterns of cognitive ageing may differ across countries and populations, and replication in other cultural and demographic settings will help establish the broader applicability of these findings.

## 5. Conclusions

In conclusion, cMIND, dietary diversity, and HPDI were all positively associated with global cognitive function in older Chinese adults, with associations also evident for specific cognitive domains. The observed dose–response patterns and age-related heterogeneity further illustrate the complexity of diet–cognition relationships across later life. These findings provide a multidimensional perspective on the relationship between diet and cognitive health and highlight the importance of considering dietary dimensions, cognitive domains, and age when studying cognitive ageing.

## Figures and Tables

**Table 1:** Characteristics of participants included in the analysis.

Characteristic	Category	Overall (N = 9615)
Age, years	-	84.62 ± 10.80
Age group	65–74 years	2023 (21.0%)
	75–84 years	3000 (31.2%)
	85–99 years	3462 (36.0%)
	100 years or older	1130 (11.8%)
Sex	Male	4453 (46.3%)
	Female	5162 (53.7%)
Residence	Rural	2941 (30.6%)
	Urban	6674 (69.4%)
Marital status	Other	5546 (57.8%)
	Married and living with spouse	4055 (42.2%)
Education	0 years	4622 (48.9%)
	1–6 years	3055 (32.3%)
	More than 6 years	1781 (18.8%)
Number of chronic diseases	0	2946 (31.1%)
	1	2846 (30.0%)
	2 or more	3695 (38.9%)
ADL disability	No disability	6877 (71.5%)
	Disability	2738 (28.5%)
Pension/retirement income	No	2444 (25.4%)
	Yes	7164 (74.6%)
Medical insurance	No	491 (5.1%)
	Yes	9117 (94.9%)
Self-rated economic status	Rich or very rich	1421 (14.8%)
	Average	7092 (74.0%)
	Poor or very poor	1069 (11.2%)
Current smoking	No	7935 (82.5%)
	Yes	1680 (17.5%)
Current drinking	No	8315 (86.5%)
	Yes	1300 (13.5%)
Current regular exercise	No	5740 (59.7%)
	Yes	3875 (40.3%)
Social participation	No	8189 (85.2%)
	Yes	1425 (14.8%)
BMI, kg/m^2^	-	23.24 ± 6.37
Nine-item depressive symptom score (CESD-9)	-	10.04 ± 5.81
cMIND	-	4.23 ± 1.62
Dietary diversity	-	4.16 ± 2.03
HPDI	-	5.31 ± 1.96
Global cognitive function	-	21.57 ± 10.17
Orientation	-	3.86 ± 1.86
Memory	-	4.25 ± 2.37
Attention, executive, and visuospatial function	-	8.73 ± 4.49
Language	-	4.73 ± 2.18
Hypertension	No	5451 (57.7%)
	Yes	3989 (42.3%)
Diabetes	No	8428 (89.5%)
	Yes	985 (10.5%)
Heart disease	No	7776 (82.8%)
	Yes	1612 (17.2%)
Stroke/cerebrovascular disease	No	8341 (88.8%)
	Yes	1047 (11.2%)
Parkinson’s disease	No	9205 (98.6%)
	Yes	135 (1.4%)
Dementia	No	9018 (95.6%)
	Yes	411 (4.4%)

Notes: Values are presented as N (%) for categorical variables and mean ± SD for continuous variables. Percentages were calculated among participants with non-missing data for the corresponding variable. Descriptive statistics are unweighted and based on the original data before multiple imputation. Missing covariate data were subsequently handled using multiple imputation by chained equations (20 imputed datasets; 30 iterations). ADL, activities of daily living; BMI, body mass index; CESD-9, nine-item Center for Epidemiologic Studies Depression score; cMIND, Chinese MIND diet; HPDI, healthy plant-based diet index; SD, standard deviation.

**Table 2:** Associations of dietary indices with global and domain-specific cognitive function.

Cognitive Outcome	Dietary Index	Model 1 β (95% CI)	Model 2 β (95% CI)	Model 3 β (95% CI)	Model 4 β (95% CI)
Global cognitive function	cMIND	1.170 (0.954 to 1.386) ***	0.677 (0.426 to 0.928) ***	0.323 (0.118 to 0.529) **	0.355 (0.147 to 0.563) ***
	Dietary diversity	1.018 (0.758 to 1.278) ***	0.649 (0.379 to 0.920) ***	0.323 (0.097 to 0.549) **	0.342 (0.119 to 0.565) **
	HPDI	1.236 (0.939 to 1.533) ***	0.739 (0.454 to 1.023) ***	0.367 (0.105 to 0.629) **	0.388 (0.132 to 0.643) **
Orientation	cMIND	0.154 (0.116 to 0.192) ***	0.090 (0.047 to 0.133) ***	0.030 (−0.006 to 0.066)	0.036 (−0.000 to 0.072)
	Dietary diversity	0.142 (0.097 to 0.187) ***	0.096 (0.056 to 0.137) ***	0.042 (0.006 to 0.077) *	0.045 (0.010 to 0.080) *
	HPDI	0.180 (0.126 to 0.234) ***	0.113 (0.064 to 0.161) ***	0.052 (0.005 to 0.099) *	0.055 (0.009 to 0.101) *
Memory	cMIND	0.182 (0.127 to 0.237) ***	0.105 (0.043 to 0.166) ***	0.043 (−0.012 to 0.097)	0.047 (−0.008 to 0.102)
	Dietary diversity	0.129 (0.062 to 0.196) ***	0.074 (0.002 to 0.145) *	0.016 (−0.046 to 0.078)	0.020 (−0.042 to 0.081)
	HPDI	0.208 (0.134 to 0.282) ***	0.129 (0.057 to 0.200) ***	0.060 (−0.008 to 0.129)	0.065 (−0.002 to 0.133)
Attention, executive, and visuospatial function	cMIND	0.677 (0.562 to 0.792) ***	0.381 (0.255 to 0.508) ***	0.221 (0.111 to 0.331) ***	0.238 (0.126 to 0.349) ***
	Dietary diversity	0.619 (0.488 to 0.751) ***	0.392 (0.250 to 0.533) ***	0.243 (0.114 to 0.373) ***	0.252 (0.123 to 0.381) ***
	HPDI	0.690 (0.547 to 0.834) ***	0.396 (0.252 to 0.539) ***	0.227 (0.090 to 0.365) **	0.237 (0.102 to 0.371) ***
Language	cMIND	0.157 (0.118 to 0.196) ***	0.101 (0.052 to 0.150) ***	0.029 (−0.011 to 0.069)	0.034 (−0.006 to 0.075)
	Dietary diversity	0.127 (0.074 to 0.181) ***	0.088 (0.030 to 0.146) **	0.022 (−0.023 to 0.067)	0.025 (−0.020 to 0.071)
	HPDI	0.158 (0.096 to 0.220) ***	0.102 (0.041 to 0.162) ***	0.028 (−0.025 to 0.080)	0.031 (−0.021 to 0.083)

Notes: β coefficients represent adjusted differences in cognitive scores associated with a 1-SD higher dietary index. Model 1 was unadjusted. Model 2 adjusted for age group, sex, residence, marital status, education, pension/retirement income, medical insurance, and self-rated economic status. Model 3 additionally adjusted for number of chronic diseases, ADL disability, current smoking, current drinking, regular exercise, social participation, depressive symptoms, and BMI. Model 4 replaced the number of chronic diseases with hypertension, diabetes, heart disease, stroke/cerebrovascular disease, and Parkinson’s disease. All models were weighted using the 2021 sampling weight. * *p* < 0.05; ** *p* < 0.01; *** *p* < 0.001. ADL, activities of daily living; BMI, body mass index; CI, confidence interval; SD, standard deviation.

**Table 3:** Interactions between dietary indices and age in relation to global cognitive function.

Dietary Index	Diet × Age Interaction β (95% CI)
cMIND	0.049 (0.032 to 0.065) ***
Dietary diversity	0.028 (0.013 to 0.044) ***
HPDI	0.043 (0.025 to 0.061) ***

Notes: β coefficients represent the change in the association between a 1-point higher dietary index score and global cognitive function for each 1-year increase in age. Age was modeled continuously and centered at 80 years. Models were weighted and fully adjusted as described in the Methods. *** *p*-interaction < 0.001. CI, confidence interval.

**Table 4:** Sensitivity analysis after excluding participants with age-specific very low MMSE scores or reported dementia.

Cognitive Outcome	Dietary Index	Model 5 β (95% CI)
Global cognitive function	cMIND	0.262 (0.161 to 0.362) ***
Orientation	cMIND	0.023 (0.008 to 0.038) **
Memory	cMIND	0.038 (−0.003 to 0.078)
Attention, executive, and visuospatial function	cMIND	0.168 (0.094 to 0.243) ***
Language	cMIND	0.033 (0.018 to 0.048) ***
Global cognitive function	Dietary diversity	0.199 (0.068 to 0.329) **
Orientation	Dietary diversity	0.014 (−0.002 to 0.030)
Memory	Dietary diversity	−0.003 (−0.043 to 0.037)
Attention, executive, and visuospatial function	Dietary diversity	0.161 (0.063 to 0.259) ***
Language	Dietary diversity	0.027 (0.014 to 0.040) ***
Global cognitive function	HPDI	0.218 (0.094 to 0.341) ***
Orientation	HPDI	0.019 (0.003 to 0.035) *
Memory	HPDI	0.034 (−0.005 to 0.073)
Attention, executive, and visuospatial function	HPDI	0.136 (0.044 to 0.228) **
Language	HPDI	0.029 (0.013 to 0.045) ***

Notes: Model 5 excluded participants with MMSE scores at or below the age-specific weighted 5th percentile within 5-year age groups and/or reported dementia (N = 8268) and used the same covariate adjustment as Model 4 in Table 2. β coefficients represent differences in cognitive scores per 1-SD higher dietary index. Models were weighted using the 2021 sampling weight. * *p* < 0.05; ** *p* < 0.01; *** *p* < 0.001. CI, confidence interval; MMSE, Mini-Mental State Examination.

## Data Availability

The data used in this study are publicly available from the Peking University Open Research Data Platform at https://opendata.pku.edu.cn/dataverse/CHADS (accessed on 26 August 2026).

## Supplementary Materials

The following supporting information can be downloaded at: https://www.mdpi.com/article/10.3390/nu18203365/s1, Table S1: Construction and scoring of the three dietary indices; Table S2: Missing data for variables included in the analyses; Table S3: Age-specific MMSE thresholds and exclusions for the low-cognition sensitivity analysis; Table S4: Correlations among the three dietary indices; Table S5: Multivariable associations of cMIND score and covariates with global and domain-specific cognitive function; Table S6: Multivariable associations of dietary diversity score and covariates with global and domain-specific cognitive function; Table S7: Multivariable associations of HPDI and covariates with global and domain-specific cognitive function; Table S8: Associations of quartiles of dietary indices with global and domain-specific cognitive function; Table S9: Restricted cubic spline analyses of dietary indices and global and domain-specific cognitive function; Table S10: Mutually adjusted associations of the three dietary indices with cognitive outcomes and collinearity diagnostics; Table S11: Formal comparisons of standardized associations across dietary indices; Table S12: Sensitivity analysis comparing chronic-disease-count and disease-specific adjustment; Table S13: Complete-case sensitivity analysis of dietary indices and cognitive outcomes; Table S14: Sensitivity analysis of diet-by-age interactions after excluding participants with age-specific very low MMSE scores or reported dementia; Figure S1: Correlations among the three dietary indices; Figure S2: Dose–response associations of cMIND score with cognitive function after excluding participants with age-specific very low MMSE scores or reported dementia; Figure S3: Dose–response associations of dietary diversity with cognitive function after excluding participants with age-specific very low MMSE scores or reported dementia; Figure S4: Dose–response associations of HPDI with cognitive function after excluding participants with age-specific very low MMSE scores or reported dementia; Figure S5: Age-related heterogeneity in associations between dietary indices and global cognitive function after excluding participants with age-specific very low MMSE scores or reported dementia.

## Abbreviations

ADL, activities of daily living; CI, confidence interval; CLHLS, Chinese Longitudinal Healthy Longevity Survey; cMIND, Chinese MIND diet; C-MMSE, Chinese version of the Mini-Mental State Examination; DASH, Dietary Approaches to Stop Hypertension; HPDI, healthy plant-based diet index; MICE, multiple imputation by chained equations; MIND, Mediterranean-DASH Intervention for Neurodegenerative Delay; MMSE, Mini-Mental State Examination; SD, standard deviation.
