# Supplementary material for: Associations of Distinct Dietary Indices with Global and Domain-Specific Cognition and Age-Related Heterogeneity in Older Chinese Adults
Source: Nutrients. 2026 Oct 14;18(20):3365. doi: 10.3390/nu18203365 (PMC13708354; doi:10.3390/nu18203365)
Supplement: Supplementary file 1 [file nutrients-18-03365-s001.zip › nutrients-4584431-supplementary.pdf]

Supplementary appendix

Contents

Table S1. Construction and scoring of the three dietary indices 3
Table S2. Missing data for variables included in the analyses 5
Table S3. Age-specific MMSE thresholds and exclusions for the low-cognition sensitivity analysis 6
Table S4. Correlations among the three dietary indices 18
Table S5. Multivariable associations of cMIND score and covariates with global and domain-specific cognitive function 6
Table S6. Multivariable associations of dietary diversity score and covariates with global and domain-specific cognitive function 10
Table S7. Multivariable associations of HPDI and covariates with global and domain-specific cognitive function 14
Table S8. Associations of quartiles of dietary indices with global and domain-specific cognitive function 18
Table S9. Restricted cubic spline analyses of dietary indices and global and domain-specific cognitive function 24
Table S10. Mutually adjusted associations of the three dietary indices with cognitive outcomes and collinearity diagnostics 21
Table S11. Formal comparisons of standardized associations across dietary indices 23
Table S12. Sensitivity analysis comparing chronic-disease-count and disease-specific adjustment 24
Table S13. Complete-case sensitivity analysis of dietary indices and cognitive outcomes 26
Table S14. Sensitivity analysis of diet-by-age interactions after excluding participants with age-specific very low MMSE scores or reported dementia 27
Figure S1. Correlations among the three dietary indices 29
Figure S2. Dose–response associations of cMIND score with cognitive function after excluding participants with age-specific very low MMSE scores or reported dementia 30
Figure S3. Dose–response associations of dietary diversity with cognitive function after excluding participants with age-specific very low MMSE scores or reported dementia 31

Figure S4. Dose–response associations of HPDI with cognitive function after excluding participants with age-specific very low MMSE scores or reported dementia32
Figure S5. Age-related heterogeneity in associations between dietary indices and global cognitive function after excluding participants with age-specific very low MMSE scores or reported dementia... 33

Table S1. Construction and scoring of the three dietary indices

Dietary patterns	Scoring
cMIND	
**Type of staple food**	0 = rice or wheat; 1 = whole grains
**Amount of staple food**	0 = <250 or >400 g/day; 1 = 250–400 g/day
**Fresh vegetables**	0 = ≤2 servings/week; 0.5 = 3–5 servings/week; 1 = ≥6 servings/week
**Mushroom or algae**	0 = ≤1 meal/week; 0.5 = 1–3 meals/week; 1 = ≥4 meals/week
**Fresh fruit**	0 = ≤2 servings/week; 0.5 = 3–5 servings/week; 1 = ≥6 servings/week
**Cooking oil**	0 = animal oil; 1 = vegetable oil
**Fish**	0 = <1 meal/month; 0.5 = 1–3 meals/month; 1 = ≥1 meal/week
**Soybeans**	0 = <1 meal/week; 0.5 = 1–3 meals/week; 1 = ≥4 meals/week
**Nuts**	0 = <1 serving/week; 0.5 = 1–4 servings/week; 1 = ≥5 servings/week
**Garlic**	0 = <1 meal/week; 0.5 = 1–3 meals/week; 1 = ≥4 meals/week
**Tea**	0 = not almost every day; 0.5 = other types of tea almost every day; 1 = green tea almost every day
**Sugar or sweets**	0 = ≥2 servings/week; 0.5 = 1 serving/month to 1 serving/week; 1 = <1 serving/month
Dietary diversity score	
**Fruits**	0 = not consumed daily; 1 = consumed daily
**Vegetables**	0 = not consumed daily; 1 = consumed daily
**Grains**	0 = not consumed daily; 1 = consumed daily
**Meat**	0 = not consumed daily; 1 = consumed daily
**Fish and seafood**	0 = not consumed daily; 1 = consumed daily
**Eggs**	0 = not consumed daily; 1 = consumed daily
**Dairy products**	0 = not consumed daily; 1 = consumed daily
**Legumes**	0 = not consumed daily; 1 = consumed daily

**Sweets**	0 = not consumed daily; 1 = consumed daily
**Garlic**	0 = not consumed daily; 1 = consumed daily
**Nuts**	0 = not consumed daily; 1 = consumed daily
**Tea**	0 = not consumed daily; 1 = consumed daily
HPDI	
**Fresh fruit**	0 = rarely or never; 1 = sometimes or occasionally; 2 = always or almost every day
**Fresh vegetables**	0 = rarely or never; 1 = sometimes or occasionally; 2 = always or almost every day
**Bean products**	0 = rarely or never; 1 = sometimes or occasionally; 2 = always or almost every day
**Garlic**	0 = rarely or never; 1 = sometimes or occasionally; 2 = always or almost every day
**Tea**	0 = rarely or never; 1 = sometimes or occasionally; 2 = always or almost every day

Abbreviations: cMIND, Chinese version of the Mediterranean-DASH Intervention for Neurodegenerative Delay diet; HPDI, healthy plant-based diet index.

Table S2. Missing data for variables included in the analyses

Variable	Missing, n	Missing, %
Education	157	1.6
Marital status	14	0.1
Number of chronic diseases	128	1.3
Pension/retirement income	7	0.1
Medical insurance	7	0.1
Nine-item depressive symptom score (CESD-9)	1,671	17.4
BMI	554	5.8
Self-rated economic status	33	0.3
Social participation	1	<0.1
Dementia	186	1.9
Hypertension	175	1.8
Diabetes	202	2.1
Heart disease	227	2.4
Stroke/cerebrovascular disease	227	2.4
Parkinson's disease	275	2.9

Notes: Missing percentages were calculated using the full analytical sample (N=9,615) as the denominator. Missing covariate data were handled using multiple imputation by chained equations. BMI, body mass index; CESD-9, nine-item Center for Epidemiologic Studies Depression score.

Table S3. Multivariable associations of cMIND score and covariates with global and domain-specific cognitive function

Variable	Level / increment	Global cognitive function	Orientation	Memory	Attention, executive, and visuospatial function	Language
**cMIND**	**Per 1 score point**	**0.225 (0.093 to 0.356)*** **	**0.023 (−0.000 to 0.046)**	**0.030 (−0.005 to 0.065)**	**0.151 (0.080 to 0.221)*** **	**0.022 (−0.004 to 0.047)**
Age group	65–74 years	Reference	Reference	Reference	Reference	Reference
	75–84 years	−0.641 (−1.105 to −0.177)**	−0.043 (−0.124 to 0.038)	−0.141 (−0.263 to −0.020)*	−0.442 (−0.673 to −0.211)***	−0.014 (−0.107 to 0.078)
	85–99 years	−3.716 (−4.540 to −2.892)***	−0.488 (−0.634 to −0.342)***	−0.822 (−1.025 to −0.619)***	−1.906 (−2.292 to −1.519)***	−0.500 (−0.671 to −0.329)***
	≥100 years	−11.278 (−12.890 to −9.665)***	−1.949 (−2.247 to −1.652)***	−2.431 (−2.836 to −2.025)***	−4.702 (−5.396 to −4.009)***	−2.195 (−2.547 to −1.843)***
Sex	Male	Reference	Reference	Reference	Reference	Reference
	Female	−0.076 (−0.672 to 0.521)	0.006 (−0.098 to 0.110)	0.041 (−0.107 to 0.188)	−0.159 (−0.449 to 0.131)	0.037 (−0.079 to 0.153)
Residence	Rural	Reference	Reference	Reference	Reference	Reference
	Urban	−0.292 (−0.701 to 0.116)	−0.102 (−0.176 to −0.029)**	−0.103 (−0.216 to 0.009)	−0.056 (−0.274 to 0.162)	−0.031 (−0.109 to 0.048)
Marital status	Other	Reference	Reference	Reference	Reference	Reference
	Married and living with spouse	0.080 (−0.361 to 0.521)	−0.021 (−0.101 to 0.058)	0.057 (−0.063 to 0.176)	−0.012 (−0.250 to 0.226)	0.057 (−0.029 to 0.142)
Education	0 years	Reference	Reference	Reference	Reference	Reference
	1–6 years	1.357 (0.793 to 1.921)***	0.224 (0.120 to 0.328)***	0.110 (−0.033 to 0.252)	0.910 (0.606 to 1.214)***	0.114 (0.013 to 0.215)*

	>6 years	1.586 (0.829 to 2.343)***	0.197 (0.063 to 0.332)**	0.202 (0.022 to 0.382)*	1.125 (0.752 to 1.498)***	0.062 (−0.084 to 0.208)
Pension/retirement income	No	Reference	Reference	Reference	Reference	Reference
	Yes	−0.553 (−1.054 to −0.051)*	−0.098 (−0.191 to −0.006)*	−0.201 (−0.333 to −0.068)**	−0.184 (−0.451 to 0.084)	−0.069 (−0.168 to 0.030)
Medical insurance	No	Reference	Reference	Reference	Reference	Reference
	Yes	0.129 (−0.826 to 1.083)	0.085 (−0.088 to 0.259)	−0.051 (−0.315 to 0.212)	0.096 (−0.438 to 0.630)	−0.001 (−0.151 to 0.148)
Self-rated economic status	Rich or very rich	Reference	Reference	Reference	Reference	Reference
	Average	−0.456 (−0.920 to 0.009)	−0.018 (−0.110 to 0.075)	−0.123 (−0.257 to 0.010)	−0.239 (−0.482 to 0.005)	−0.076 (−0.159 to 0.007)
	Poor or very poor	−1.471 (−2.509 to −0.433)**	−0.245 (−0.451 to −0.039)*	−0.406 (−0.678 to −0.134)**	−0.677 (−1.193 to −0.162)*	−0.143 (−0.330 to 0.044)
ADL disability	No disability	Reference	Reference	Reference	Reference	Reference
	Disability	−7.223 (−8.944 to −5.502)***	−1.104 (−1.404 to −0.803)***	−1.433 (−1.834 to −1.032)***	−3.132 (−3.871 to −2.393)***	−1.554 (−1.932 to −1.177)***
Current smoking	No	Reference	Reference	Reference	Reference	Reference
	Yes	−0.014 (−0.568 to 0.540)	0.041 (−0.047 to 0.129)	0.025 (−0.134 to 0.185)	−0.081 (−0.388 to 0.226)	0.001 (−0.122 to 0.123)
Current drinking	No	Reference	Reference	Reference	Reference	Reference
	Yes	0.253 (−0.247 to 0.752)	0.041 (−0.040 to 0.122)	0.010 (−0.145 to 0.165)	0.135 (−0.155 to 0.425)	0.067 (−0.032 to 0.165)
Current regular exercise	No	Reference	Reference	Reference	Reference	Reference

	Yes	0.449 (0.037 to 0.861)*	0.125 (0.053 to 0.198)***	0.096 (−0.022 to 0.213)	0.095 (−0.139 to 0.329)	0.133 (0.050 to 0.216)**
Social participation	No	Reference	Reference	Reference	Reference	Reference
	Yes	0.714 (0.352 to 1.075)***	0.094 (0.040 to 0.149)***	0.139 (0.028 to 0.251)*	0.422 (0.199 to 0.645)***	0.058 (−0.010 to 0.126)
Nine-item depressive symptom score (CESD-9)	Per 1 score point	−0.069 (−0.124 to −0.014)*	−0.011 (−0.021 to −0.002)*	−0.006 (−0.019 to 0.007)	−0.041 (−0.068 to −0.014)**	−0.011 (−0.023 to 0.001)
BMI, kg/m²	Per 1 kg/m²	0.041 (0.006 to 0.077)*	0.007 (0.001 to 0.014)*	0.007 (−0.002 to 0.017)	0.020 (0.001 to 0.039)*	0.007 (0.000 to 0.013)*
Hypertension	No	Reference	Reference	Reference	Reference	Reference
	Yes	−0.160 (−0.588 to 0.268)	−0.025 (−0.100 to 0.050)	−0.036 (−0.155 to 0.082)	−0.086 (−0.302 to 0.131)	−0.014 (−0.101 to 0.074)
Diabetes	No	Reference	Reference	Reference	Reference	Reference
	Yes	−0.307 (−0.995 to 0.381)	−0.054 (−0.183 to 0.075)	−0.044 (−0.215 to 0.127)	−0.163 (−0.526 to 0.200)	−0.046 (−0.179 to 0.086)
Heart disease	No	Reference	Reference	Reference	Reference	Reference
	Yes	1.386 (0.786 to 1.987)***	0.188 (0.085 to 0.290)***	0.242 (0.089 to 0.396)**	0.697 (0.396 to 0.998)***	0.259 (0.147 to 0.371)***
Stroke/cerebrovascular disease	No	Reference	Reference	Reference	Reference	Reference
	Yes	−0.696 (−1.504 to 0.112)	−0.013 (−0.152 to 0.126)	−0.263 (−0.483 to −0.043)*	−0.318 (−0.738 to 0.101)	−0.102 (−0.267 to 0.064)
Parkinson's disease	No	Reference	Reference	Reference	Reference	Reference
	Yes	−2.631 (−4.933 to	−0.561 (−1.021 to	−0.603 (−1.189 to	−1.030 (−2.082 to 0.022)	−0.437 (−1.013 to

	−0.330)*	−0.102)*	−0.016)*		0.139)

Notes: Values are β coefficients (95% CIs) from Model 4. The cMIND score and all covariates shown were included simultaneously in each model. The cMIND association is reported per 1-point increment. Models were weighted using the 2021 sampling weight. *P<0.05; **P<0.01; ***P<0.001. ADL, activities of daily living; BMI, body mass index; CESD-9, nine-item Center for Epidemiologic Studies Depression score; CI, confidence interval.

Table S4. Multivariable associations of dietary diversity score and covariates with global and domain-specific cognitive function

Variable	Level / increment	Global cognitive function	Orientation	Memory	Attention, executive, and visuospatial function	Language
Dietary diversity	Per 1 score point	0.168 (0.058 to 0.278)**	0.022 (0.005 to 0.039)*	0.010 (−0.020 to 0.040)	0.124 (0.061 to 0.187)***	0.012 (−0.010 to 0.035)
Age group	65–74 years	Reference	Reference	Reference	Reference	Reference
	75–84 years	−0.704 (−1.168 to −0.239)**	−0.051 (−0.131 to 0.030)	−0.147 (−0.269 to −0.025)*	−0.486 (−0.716 to −0.256)***	−0.020 (−0.113 to 0.074)
	85–99 years	−3.792 (−4.613 to −2.971)***	−0.497 (−0.642 to −0.352)***	−0.829 (−1.032 to −0.626)***	−1.959 (−2.343 to −1.575)***	−0.507 (−0.679 to −0.335)***
	≥100 years	−11.390 (−13.003 to −9.777)***	−1.963 (−2.259 to −1.666)***	−2.441 (−2.848 to −2.035)***	−4.782 (−5.474 to −4.089)***	−2.204 (−2.558 to −1.850)***
Sex	Male	Reference	Reference	Reference	Reference	Reference
	Female	−0.110 (−0.708 to 0.487)	0.002 (−0.102 to 0.106)	0.037 (−0.110 to 0.185)	−0.183 (−0.472 to 0.106)	0.034 (−0.083 to 0.150)
Residence	Rural	Reference	Reference	Reference	Reference	Reference
	Urban	−0.308 (−0.715 to 0.099)	−0.106 (−0.180 to −0.033)**	−0.100 (−0.212 to 0.013)	−0.072 (−0.288 to 0.144)	−0.030 (−0.110 to 0.050)
Marital status	Other	Reference	Reference	Reference	Reference	Reference
	Married and living with spouse	0.101 (−0.339 to 0.541)	−0.020 (−0.099 to 0.060)	0.060 (−0.059 to 0.179)	0.002 (−0.235 to 0.238)	0.059 (−0.027 to 0.145)
Education	0 years	Reference	Reference	Reference	Reference	Reference
	1–6 years	1.367 (0.809 to	0.223 (0.120 to	0.115 (−0.028 to	0.913 (0.617 to	0.116 (0.015 to

		1.925)***	0.327)***	0.258)	1.208)***	0.217)*
	>6 years	1.583 (0.833 to 2.334)***	0.193 (0.061 to 0.324)**	0.212 (0.032 to 0.392)*	1.113 (0.745 to 1.482)***	0.065 (−0.080 to 0.210)
Pension/retirement income	No	Reference	Reference	Reference	Reference	Reference
	Yes	−0.561 (−1.056 to −0.066)*	−0.100 (−0.192 to −0.008)*	−0.200 (−0.332 to −0.067)**	−0.191 (−0.454 to 0.071)	−0.070 (−0.169 to 0.030)
Medical insurance	No	Reference	Reference	Reference	Reference	Reference
	Yes	0.155 (−0.798 to 1.107)	0.086 (−0.090 to 0.262)	−0.042 (−0.305 to 0.221)	0.108 (−0.427 to 0.643)	0.003 (−0.147 to 0.153)
Self-rated economic status	Rich or very rich	Reference	Reference	Reference	Reference	Reference
	Average	−0.458 (−0.924 to 0.009)	−0.017 (−0.110 to 0.077)	−0.128 (−0.262 to 0.007)	−0.236 (−0.480 to 0.007)	−0.077 (−0.162 to 0.007)
	Poor or very poor	−1.471 (−2.473 to −0.468)**	−0.241 (−0.441 to −0.041)*	−0.416 (−0.683 to −0.148)**	−0.668 (−1.168 to −0.168)**	−0.146 (−0.327 to 0.035)
ADL disability	No disability	Reference	Reference	Reference	Reference	Reference
	Disability	−7.264 (−8.981 to −5.547)***	−1.107 (−1.406 to −0.808)***	−1.440 (−1.840 to −1.040)***	−3.158 (−3.894 to −2.422)***	−1.559 (−1.936 to −1.181)***
Current smoking	No	Reference	Reference	Reference	Reference	Reference
	Yes	−0.078 (−0.627 to 0.471)	0.033 (−0.054 to 0.120)	0.020 (−0.138 to 0.177)	−0.126 (−0.429 to 0.176)	−0.004 (−0.125 to 0.116)
Current drinking	No	Reference	Reference	Reference	Reference	Reference
	Yes	0.206 (−0.278 to 0.690)	0.033 (−0.047 to 0.112)	0.012 (−0.145 to 0.170)	0.096 (−0.179 to 0.370)	0.065 (−0.037 to 0.167)

Current regular exercise	No	Reference	Reference	Reference	Reference	Reference
	Yes	0.482 (0.070 to 0.893)*	0.127 (0.055 to 0.199)***	0.105 (−0.011 to 0.220)	0.113 (−0.119 to 0.345)	0.137 (0.055 to 0.219)**
Social participation	No	Reference	Reference	Reference	Reference	Reference
	Yes	0.719 (0.352 to 1.086)***	0.093 (0.039 to 0.148)***	0.144 (0.030 to 0.258)*	0.422 (0.197 to 0.647)***	0.060 (−0.010 to 0.129)
Nine-item depressive symptom score (CESD-9)	Per 1 score point	−0.065 (−0.120 to −0.009)*	−0.011 (−0.021 to −0.001)*	−0.006 (−0.019 to 0.007)	−0.037 (−0.065 to −0.010)**	−0.011 (−0.022 to 0.001)
BMI, kg/m²	Per 1 kg/m²	0.044 (0.008 to 0.079)*	0.008 (0.001 to 0.014)*	0.008 (−0.002 to 0.017)	0.021 (0.002 to 0.040)*	0.007 (0.000 to 0.014)*
Hypertension	No	Reference	Reference	Reference	Reference	Reference
	Yes	−0.124 (−0.547 to 0.298)	−0.021 (−0.095 to 0.053)	−0.031 (−0.149 to 0.086)	−0.062 (−0.276 to 0.152)	−0.010 (−0.097 to 0.076)
Diabetes	No	Reference	Reference	Reference	Reference	Reference
	Yes	−0.235 (−0.928 to 0.457)	−0.047 (−0.177 to 0.082)	−0.033 (−0.204 to 0.139)	−0.116 (−0.483 to 0.250)	−0.039 (−0.172 to 0.094)
Heart disease	No	Reference	Reference	Reference	Reference	Reference
	Yes	1.376 (0.773 to 1.978)***	0.186 (0.084 to 0.289)***	0.241 (0.088 to 0.395)**	0.690 (0.388 to 0.992)***	0.258 (0.146 to 0.371)***
Stroke/cerebrovascular disease	No	Reference	Reference	Reference	Reference	Reference
	Yes	−0.711 (−1.520 to 0.099)	−0.015 (−0.153 to 0.124)	−0.264 (−0.485 to −0.042)*	−0.330 (−0.749 to 0.090)	−0.103 (−0.268 to 0.063)
Parkinson's disease	No	Reference	Reference	Reference	Reference	Reference

Yes	−2.656 (−4.953 to −0.359)*	−0.566 (−1.022 to −0.111)*	−0.600 (−1.183 to −0.016)*	−1.053 (−2.107 to 0.002)	−0.438 (−1.014 to 0.139)

Values are β coefficients (95% CIs) from Model 4. The dietary diversity score and all covariates shown were included simultaneously in each model. The dietary diversity association is reported per 1-point increment. Models were weighted using the 2021 sampling weight. *P<0.05; **P<0.01; ***P<0.001. ADL, activities of daily living; BMI, body mass index; CESD-9, nine-item Center for Epidemiologic Studies Depression score; CI, confidence interval.

Table S5. Multivariable associations of HPDI and covariates with global and domain-specific cognitive function

Variable	Level / increment	Global cognitive function	Orientation	Memory	Attention, executive, and visuospatial function	Language
**HPDI**	**Per 1 score point**	**0.212 (0.072 to 0.351)****	**0.030 (0.005 to 0.055)***	**0.036 (−0.001 to 0.072)**	**0.129 (0.056 to 0.202)*****	**0.017 (−0.011 to 0.045)**
Age group	65–74 years	Reference	Reference	Reference	Reference	Reference
	75–84 years	−0.654 (−1.118 to −0.189)**	−0.044 (−0.125 to 0.037)	−0.143 (−0.264 to −0.021)*	−0.451 (−0.682 to −0.220)***	−0.016 (−0.108 to 0.076)
	85–99 years	−3.719 (−4.543 to −2.896)***	−0.487 (−0.634 to −0.341)***	−0.821 (−1.024 to −0.618)***	−1.910 (−2.296 to −1.523)***	−0.501 (−0.672 to −0.330)***
	≥100 years	−11.270 (−12.884 to −9.656)***	−1.946 (−2.244 to −1.648)***	−2.428 (−2.834 to −2.022)***	−4.701 (−5.395 to −4.007)***	−2.195 (−2.547 to −1.843)***
Sex	Male	Reference	Reference	Reference	Reference	Reference
	Female	−0.041 (−0.655 to 0.572)	0.012 (−0.095 to 0.118)	0.047 (−0.103 to 0.198)	−0.139 (−0.436 to 0.157)	0.039 (−0.080 to 0.159)
Residence	Rural	Reference	Reference	Reference	Reference	Reference
	Urban	−0.258 (−0.659 to 0.144)	−0.100 (−0.172 to −0.028)**	−0.100 (−0.211 to 0.011)	−0.031 (−0.248 to 0.186)	−0.027 (−0.104 to 0.051)
Marital status	Other	Reference	Reference	Reference	Reference	Reference
	Married and living with spouse	0.084 (−0.358 to 0.526)	−0.022 (−0.102 to 0.058)	0.056 (−0.064 to 0.176)	−0.008 (−0.247 to 0.232)	0.058 (−0.028 to 0.143)
Education	0 years	Reference	Reference	Reference	Reference	Reference
	1–6 years	1.374 (0.817 to	0.224 (0.121 to	0.110 (−0.032 to	0.924 (0.626 to	0.116 (0.014 to

		1.931)***	0.326)***	0.252)	1.221)***	0.218)*
	>6 years	1.604 (0.873 to 2.335)***	0.194 (0.066 to 0.322)**	0.200 (0.024 to 0.375)*	1.144 (0.781 to 1.507)***	0.066 (−0.075 to 0.207)
Pension/retirement income	No	Reference	Reference	Reference	Reference	Reference
	Yes	−0.546 (−1.040 to −0.053)*	−0.098 (−0.190 to −0.007)*	−0.200 (−0.332 to −0.069)**	−0.179 (−0.443 to 0.084)	−0.069 (−0.167 to 0.030)
Medical insurance	No	Reference	Reference	Reference	Reference	Reference
	Yes	0.126 (−0.852 to 1.104)	0.081 (−0.099 to 0.260)	−0.056 (−0.324 to 0.213)	0.101 (−0.444 to 0.646)	0.000 (−0.152 to 0.153)
Self-rated economic status	Rich or very rich	Reference	Reference	Reference	Reference	Reference
	Average	−0.487 (−0.957 to −0.016)*	−0.020 (−0.113 to 0.073)	−0.126 (−0.261 to 0.008)	−0.261 (−0.506 to −0.015)*	−0.079 (−0.164 to 0.005)
	Poor or very poor	−1.424 (−2.416 to −0.432)**	−0.233 (−0.429 to −0.037)*	−0.393 (−0.656 to −0.130)**	−0.656 (−1.154 to −0.159)**	−0.141 (−0.321 to 0.038)
ADL disability	No disability	Reference	Reference	Reference	Reference	Reference
	Disability	−7.246 (−8.976 to −5.516)***	−1.104 (−1.405 to −0.803)***	−1.435 (−1.838 to −1.031)***	−3.150 (−3.893 to −2.407)***	−1.557 (−1.936 to −1.178)***
Current smoking	No	Reference	Reference	Reference	Reference	Reference
	Yes	−0.063 (−0.617 to 0.490)	0.035 (−0.052 to 0.122)	0.018 (−0.141 to 0.177)	−0.113 (−0.420 to 0.195)	−0.004 (−0.125 to 0.118)
Current drinking	No	Reference	Reference	Reference	Reference	Reference
	Yes	0.198 (−0.293 to 0.688)	0.031 (−0.050 to 0.111)	−0.002 (−0.158 to 0.155)	0.105 (−0.175 to 0.385)	0.063 (−0.038 to 0.165)

Current regular exercise	No	Reference	Reference	Reference	Reference	Reference
	Yes	0.459 (0.039 to 0.879)*	0.123 (0.050 to 0.196)***	0.094 (−0.022 to 0.210)	0.107 (−0.128 to 0.342)	0.135 (0.052 to 0.219)**
Social participation	No	Reference	Reference	Reference	Reference	Reference
	Yes	0.681 (0.305 to 1.058)***	0.088 (0.031 to 0.144)**	0.132 (0.016 to 0.248)*	0.405 (0.177 to 0.634)***	0.056 (−0.017 to 0.129)
Nine-item depressive symptom score (CESD-9)	Per 1 score point	−0.067 (−0.122 to −0.011)*	−0.011 (−0.021 to −0.001)*	−0.005 (−0.018 to 0.008)	−0.040 (−0.067 to −0.012)**	−0.011 (−0.023 to 0.001)
BMI, kg/m²	Per 1 kg/m²	0.042 (0.006 to 0.078)*	0.007 (0.001 to 0.014)*	0.007 (−0.002 to 0.017)	0.020 (0.001 to 0.040)*	0.007 (0.000 to 0.013)*
Hypertension	No	Reference	Reference	Reference	Reference	Reference
	Yes	−0.151 (−0.573 to 0.272)	−0.025 (−0.098 to 0.049)	−0.036 (−0.153 to 0.082)	−0.078 (−0.293 to 0.137)	−0.012 (−0.098 to 0.074)
Diabetes	No	Reference	Reference	Reference	Reference	Reference
	Yes	−0.209 (−0.903 to 0.486)	−0.044 (−0.175 to 0.087)	−0.031 (−0.202 to 0.140)	−0.097 (−0.466 to 0.271)	−0.037 (−0.170 to 0.096)
Heart disease	No	Reference	Reference	Reference	Reference	Reference
	Yes	1.410 (0.808 to 2.012)***	0.191 (0.089 to 0.294)***	0.247 (0.093 to 0.400)**	0.711 (0.409 to 1.014)***	0.261 (0.150 to 0.373)***
Stroke/cerebrovascular disease	No	Reference	Reference	Reference	Reference	Reference
	Yes	−0.678 (−1.490 to 0.135)	−0.010 (−0.149 to 0.129)	−0.260 (−0.481 to −0.039)*	−0.307 (−0.729 to 0.116)	−0.100 (−0.266 to 0.065)
Parkinson's disease	No	Reference	Reference	Reference	Reference	Reference

Yes	−2.609 (−4.945 to −0.274)*	−0.561 (−1.022 to −0.099)*	−0.601 (−1.197 to −0.006)*	−1.013 (−2.079 to 0.053)	−0.434 (−1.013 to 0.144)

Notes: Values are β coefficients (95% CIs) from Model 4. The HPDI score and all covariates shown were included simultaneously in each model. The HPDI association is reported per 1-point increment. Models were weighted using the 2021 sampling weight. P<0.05; P<0.01; P<0.001. ADL, activities of daily living; BMI, body mass index; CESD-9, nine-item Center for Epidemiologic Studies Depression score; CI, confidence interval.

Table S6. Associations of quartiles of dietary indices with global and domain-specific cognitive function

Quartile	Global cognitive function	Orientation	Memory	Attention, executive, and visuospatial function	Language
Panel A. cMIND					
Q1	Reference	Reference	Reference	Reference	Reference
Q2	0.336 (−0.315, 0.988)	0.049 (−0.069, 0.167)	0.011 (−0.157, 0.178)	0.215 (−0.126, 0.557)	0.061 (−0.058, 0.181)
Q3	0.818 (0.199, 1.437)**	0.092 (−0.015, 0.199)	0.081 (−0.077, 0.240)	0.579 (0.250, 0.908)***	0.066 (−0.058, 0.190)
Q4	0.950 (0.394, 1.506)***	0.090 (−0.011, 0.191)	0.141 (−0.017, 0.300)	0.619 (0.307, 0.930)***	0.100 (−0.004, 0.204)
P for trend	**	NS	NS	***	NS
Panel B. Dietary diversity					
Q1	Reference	Reference	Reference	Reference	Reference
Q2	0.330 (−0.396, 1.057)	0.009 (−0.123, 0.142)	−0.042 (−0.223, 0.139)	0.350 (−0.001, 0.700)	0.013 (−0.127, 0.154)
Q3	0.561 (0.083, 1.038)*	0.061 (−0.019, 0.141)	0.019 (−0.110, 0.148)	0.418 (0.144, 0.692)**	0.063 (−0.026, 0.152)
Q4	0.705 (0.085, 1.325)*	0.093 (0.008, 0.178)*	0.008 (−0.175, 0.190)	0.565 (0.210, 0.921)**	0.039 (−0.097, 0.175)
P for trend	*	*	NS	***	NS
Panel C. HPDI					
Q1	Reference	Reference	Reference	Reference	Reference
Q2	0.998 (0.456, 1.541)***	0.138 (0.039, 0.238)**	0.215 (0.073, 0.357)**	0.530 (0.233, 0.828)***	0.115 (0.016, 0.214)*
Q3	0.684 (−0.170, 1.538)	0.066 (−0.083, 0.215)	0.176 (−0.029, 0.380)	0.423 (0.002, 0.845)*	0.019 (−0.146, 0.185)

Q4	0.848 (0.176, 1.520)*	0.130 (0.030, 0.229)*	0.139 (−0.049, 0.327)	0.536 (0.138, 0.935)**	0.043 (−0.093, 0.179)
P for trend	*	*	NS	**	NS

Notes: Q1 was the reference. P for trend was obtained by assigning the weighted median of each quartile and modeling it continuously. Models used Model 4 covariate adjustment and the 2021 sampling weight. CI, confidence interval.

Table S7. Correlations among the three dietary indices

Index 1	Index 2	Method	r	95% CI	P	N
cMIND	Dietary diversity	Unweighted Pearson	0.681	0.671 to 0.692	<0.001	9615
cMIND	Dietary diversity	Sampling-weighted Pearson	0.694	0.664 to 0.721	<0.001	9615
cMIND	HPDI	Unweighted Pearson	0.672	0.661 to 0.682	<0.001	9615
cMIND	HPDI	Sampling-weighted Pearson	0.685	0.661 to 0.707	<0.001	9615
Dietary diversity	HPDI	Unweighted Pearson	0.704	0.694 to 0.714	<0.001	9615
Dietary diversity	HPDI	Sampling-weighted Pearson	0.729	0.701 to 0.754	<0.001	9615

Notes: Pearson correlation coefficients are shown with 95% CIs. Weighted estimates used the 2021 sampling weight. CI, confidence interval.

Table S8. Mutually adjusted associations of the three dietary indices with cognitive outcomes and collinearity diagnostics

Panel A. Mutually adjusted associations			
Cognitive outcome	Dietary index	β (95% CI)	P
Global cognitive function	cMIND	0.156 (−0.211 to 0.522)	0.404
	Dietary diversity	0.084 (−0.223 to 0.392)	0.591
	HPDI	0.232 (−0.189 to 0.654)	0.28
Orientation	cMIND	−0.003 (−0.068 to 0.062)	0.933
	Dietary diversity	0.015 (−0.033 to 0.063)	0.537
	HPDI	0.047 (−0.031 to 0.125)	0.242
Memory	cMIND	0.030 (−0.066 to 0.126)	0.538
	Dietary diversity	−0.057 (−0.145 to 0.031)	0.203
	HPDI	0.085 (−0.020 to 0.190)	0.112
Attention, executive, and visuospatial function	cMIND	0.104 (−0.075 to 0.282)	0.255
	Dietary diversity	0.128 (−0.040 to 0.297)	0.135
	HPDI	0.084 (−0.115 to 0.283)	0.407
Language	cMIND	0.025 (−0.051 to 0.102)	0.518
	Dietary diversity	−0.002 (−0.064 to 0.060)	0.949
	HPDI	0.016 (−0.068 to 0.101)	0.702
**Panel B. Collinearity diagnostics**			
Dietary index	Minimum VIF	Median VIF	Maximum VIF
cMIND	2.403	2.409	2.414
Dietary diversity	2.723	2.727	2.733
HPDI	2.657	2.661	2.664

Notes: All three dietary indices were entered simultaneously and standardized using weighted SDs. Models otherwise used Model 4 covariate adjustment.

VIFs summarize collinearity across imputed datasets. CI, confidence interval; SD, standard deviation; VIF, variance inflation factor.

Table S9. Formal comparisons of standardized associations across dietary indices

Cognitive outcome	Comparison	Difference in β (95% CI)	P-difference	Global P
Global cognitive function	cMIND minus Dietary diversity	0.072 (-0.465 to 0.609)	0.794	0.872
Global cognitive function	cMIND minus HPDI	-0.076 (-0.799 to 0.646)	0.836	0.872
Global cognitive function	Dietary diversity minus HPDI	-0.148 (-0.738 to 0.442)	0.623	0.872
Orientation	cMIND minus Dietary diversity	-0.018 (-0.106 to 0.070)	0.692	0.762
Orientation	cMIND minus HPDI	-0.049 (-0.181 to 0.082)	0.462	0.762
Orientation	Dietary diversity minus HPDI	-0.032 (-0.136 to 0.073)	0.553	0.762
Memory	cMIND minus Dietary diversity	0.087 (-0.066 to 0.241)	0.266	0.166
Memory	cMIND minus HPDI	-0.055 (-0.234 to 0.123)	0.545	0.166
Memory	Dietary diversity minus HPDI	-0.142 (-0.296 to 0.012)	0.070	0.166
Attention, executive, and visuospatial function	cMIND minus Dietary diversity	-0.025 (-0.306 to 0.256)	0.862	0.952
Attention, executive, and visuospatial function	cMIND minus HPDI	0.019 (-0.322 to 0.361)	0.912	0.952
Attention, executive, and visuospatial function	Dietary diversity minus HPDI	0.044 (-0.246 to 0.335)	0.765	0.952
Language	cMIND minus Dietary diversity	0.027 (-0.087 to 0.142)	0.640	0.870
Language	cMIND minus HPDI	0.009 (-0.139 to 0.157)	0.908	0.870
Language	Dietary diversity minus HPDI	-0.018 (-0.134 to 0.097)	0.753	0.870

Notes: Pairwise contrasts were derived from models including all three standardized dietary indices simultaneously. Global P tests equality of the three dietary coefficients for each cognitive outcome. CI, confidence interval.

Table S10. Restricted cubic spline analyses of dietary indices and global and domain-specific cognitive function

Dietary index	Cognitive outcome	P-overall	P-nonlinearity	Knots	Reference
cMIND	Global cognitive function	0.010	0.112	2, 4, 5, 7.5	4.5
cMIND	Orientation	0.233	0.313	2, 4, 5, 7.5	4.5
cMIND	Memory	0.354	0.511	2, 4, 5, 7.5	4.5
cMIND	Attention, executive, and visuospatial function	<0.001	0.033	2, 4, 5, 7.5	4.5
cMIND	Language	0.394	0.648	2, 4, 5, 7.5	4.5
Dietary diversity	Global cognitive function	0.027	0.636	1, 3, 5, 8	4
Dietary diversity	Orientation	0.081	0.796	1, 3, 5, 8	4
Dietary diversity	Memory	0.938	0.984	1, 3, 5, 8	4
Dietary diversity	Attention, executive, and visuospatial function	0.002	0.423	1, 3, 5, 8	4
Dietary diversity	Language	0.463	0.646	1, 3, 5, 8	4
HPDI	Global cognitive function	0.001	0.006	3, 5, 6, 9	6
HPDI	Orientation	0.034	0.064	3, 5, 6, 9	6
HPDI	Memory	0.059	0.079	3, 5, 6, 9	6
HPDI	Attention, executive, and visuospatial function	<0.001	0.017	3, 5, 6, 9	6
HPDI	Language	0.011	0.005	3, 5, 6, 9	6

Notes: Four knots were placed at the weighted 5th, 35th, 65th, and 95th percentiles; the weighted median was the reference. Models used Model 4 covariate adjustment and the 2021 sampling weight.

Table S11. Sensitivity analysis comparing chronic-disease-count and disease-specific adjustment

Cognitive outcome	Dietary index	Model 3 β (95% CI)	P	Model 4 β (95% CI)	P
Global cognitive function	cMIND	0.323 (0.118 to 0.529)	0.002	0.355 (0.147 to 0.563)	<0.001
Orientation	cMIND	0.030 (-0.006 to 0.066)	0.097	0.036 (-0.000 to 0.072)	0.052
Memory	cMIND	0.043 (-0.012 to 0.097)	0.127	0.047 (-0.008 to 0.102)	0.095
Attention, executive, and visuospatial function	cMIND	0.221 (0.111 to 0.331)	<0.001	0.238 (0.126 to 0.349)	<0.001
Language	cMIND	0.029 (-0.011 to 0.069)	0.151	0.034 (-0.006 to 0.075)	0.097
Global cognitive function	Dietary diversity	0.323 (0.097 to 0.549)	0.005	0.342 (0.119 to 0.565)	0.003
Orientation	Dietary diversity	0.042 (0.006 to 0.077)	0.021	0.045 (0.010 to 0.080)	0.013
Memory	Dietary diversity	0.016 (-0.046 to 0.078)	0.607	0.020 (-0.042 to 0.081)	0.526
Attention, executive, and visuospatial function	Dietary diversity	0.243 (0.114 to 0.373)	<0.001	0.252 (0.123 to 0.381)	<0.001
Language	Dietary diversity	0.022 (-0.023 to 0.067)	0.340	0.025 (-0.020 to 0.071)	0.272
Global cognitive function	HPDI	0.367 (0.105 to 0.629)	0.006	0.388 (0.132 to 0.643)	0.003
Orientation	HPDI	0.052 (0.005 to 0.099)	0.032	0.055 (0.009 to 0.101)	0.020
Memory	HPDI	0.060 (-0.008 to 0.129)	0.083	0.065 (-0.002 to 0.133)	0.057
Attention, executive, and visuospatial function	HPDI	0.227 (0.090 to 0.365)	0.001	0.237 (0.102 to 0.371)	<0.001
Language	HPDI	0.028 (-0.025 to 0.080)	0.304	0.031 (-0.021 to 0.083)	0.239

Notes: Model 3 adjusted for chronic disease count; Model 4 replaced the count with hypertension, diabetes, heart disease, stroke/cerebrovascular disease, and Parkinson's disease. Other covariates were unchanged. CI, confidence interval.

Table S12. Complete-case sensitivity analysis of dietary indices and cognitive outcomes

Cognitive outcome	Dietary index	MICE Model 4 β (95% CI)	P	Complete-case β (95% CI)	P
Global cognitive function	cMIND	0.355 (0.147 to 0.563)	<0.001	0.320 (0.203 to 0.436)	<0.001
Orientation	cMIND	0.036 (-0.000 to 0.072)	0.052	0.029 (0.011 to 0.046)	0.002
Memory	cMIND	0.047 (-0.008 to 0.102)	0.095	0.039 (-0.004 to 0.082)	0.076
Attention, executive, and visuospatial function	cMIND	0.238 (0.126 to 0.349)	<0.001	0.217 (0.128 to 0.306)	<0.001
Language	cMIND	0.034 (-0.006 to 0.075)	0.097	0.035 (0.018 to 0.051)	<0.001
Global cognitive function	Dietary diversity	0.342 (0.119 to 0.565)	0.003	0.303 (0.136 to 0.470)	<0.001
Orientation	Dietary diversity	0.045 (0.010 to 0.080)	0.013	0.033 (0.008 to 0.057)	0.008
Memory	Dietary diversity	0.020 (-0.042 to 0.081)	0.526	0.025 (-0.021 to 0.070)	0.288
Attention, executive, and visuospatial function	Dietary diversity	0.252 (0.123 to 0.381)	<0.001	0.219 (0.094 to 0.344)	<0.001
Language	Dietary diversity	0.025 (-0.020 to 0.071)	0.272	0.027 (0.011 to 0.042)	<0.001
Global cognitive function	HPDI	0.388 (0.132 to 0.643)	0.003	0.337 (0.150 to 0.524)	<0.001
Orientation	HPDI	0.055 (0.009 to 0.101)	0.020	0.041 (0.002 to 0.079)	0.038
Memory	HPDI	0.065 (-0.002 to 0.133)	0.057	0.060 (0.008 to 0.111)	0.024
Attention, executive, and visuospatial function	HPDI	0.237 (0.102 to 0.371)	<0.001	0.210 (0.086 to 0.334)	<0.001
Language	HPDI	0.031 (-0.021 to 0.083)	0.239	0.027 (0.007 to 0.047)	0.009

Notes: Complete-case analyses included 7,289 participants with complete Model 4 covariates and used the same weighted SD scaling and covariate adjustment as the primary analysis. MICE, multiple imputation by chained equations; CI, confidence interval.

Table S13. Age-specific MMSE thresholds and exclusions for the low-cognition sensitivity analysis

Panel A. Age-specific MMSE thresholds			
Age group, years	N	Weighted MMSE 5th percentile	N with low MMSE
65–69	728	23	55
70–74	1,295	18	53
75–79	1,411	20	96
80–84	1,589	2	83
85–89	1,422	0	128
90–94	1,153	0	182
95–99	887	0	201
100–104	819	0	261
≥105	311	0	117

Panel B. Overall exclusions	
Criterion	N
Original sample	9,615
Low MMSE	1,176
Reported dementia	411
Both low MMSE and reported dementia	240
Low MMSE only	936
Reported dementia only	171
Excluded by either criterion	1,347
Final sensitivity-analysis sample	8,268

Notes: Low MMSE was defined as a score at or below the weighted 5th percentile within each 5-year age group. Because MMSE scores are discrete, ties at the thresholds resulted in more than 5% of participants meeting the low-MMSE criterion. MMSE, Mini-Mental State Examination.

Table S14. Sensitivity analysis of diet-by-age interactions after excluding participants with age-specific very low MMSE scores or reported dementia

Dietary index	Diet × age interaction β (95% CI)	P-interaction
cMIND	0.042 (0.033 to 0.052)	<0.001
Dietary diversity	0.025 (0.016 to 0.033)	<0.001
HPDI	0.034 (0.025 to 0.044)	<0.001

Notes: The sensitivity sample included 8,268 participants after excluding individuals with age-specific very low MMSE scores and/or reported dementia. β coefficients represent the change in the association between a 1-point higher dietary index score and global cognitive function for each 1-year increase in age. Age was modeled continuously and centered at 80 years. Models were weighted using the 2021 sampling weight and adjusted using the Model 4 covariates. CI, confidence interval; MMSE, Mini-Mental State Examination.

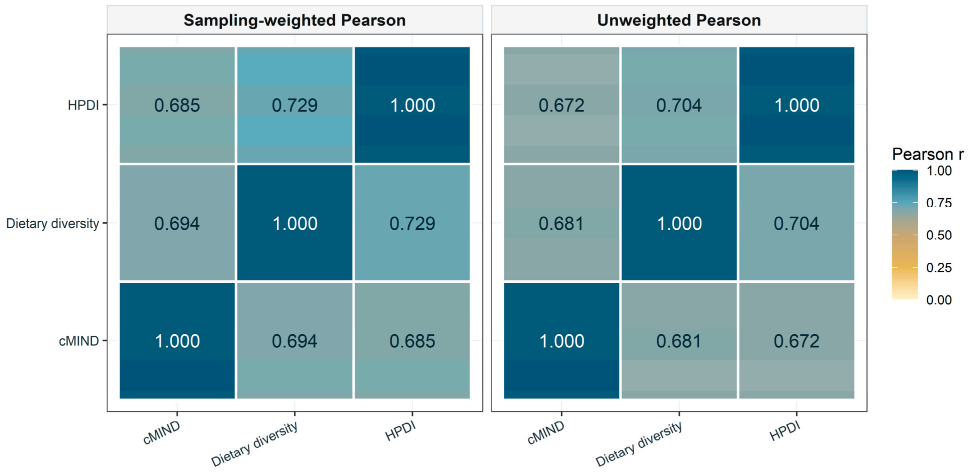

Figure S1. Correlations among the three dietary indices

Heatmaps show sampling-weighted and unweighted Pearson correlation coefficients among cMIND, dietary diversity, and HPDI

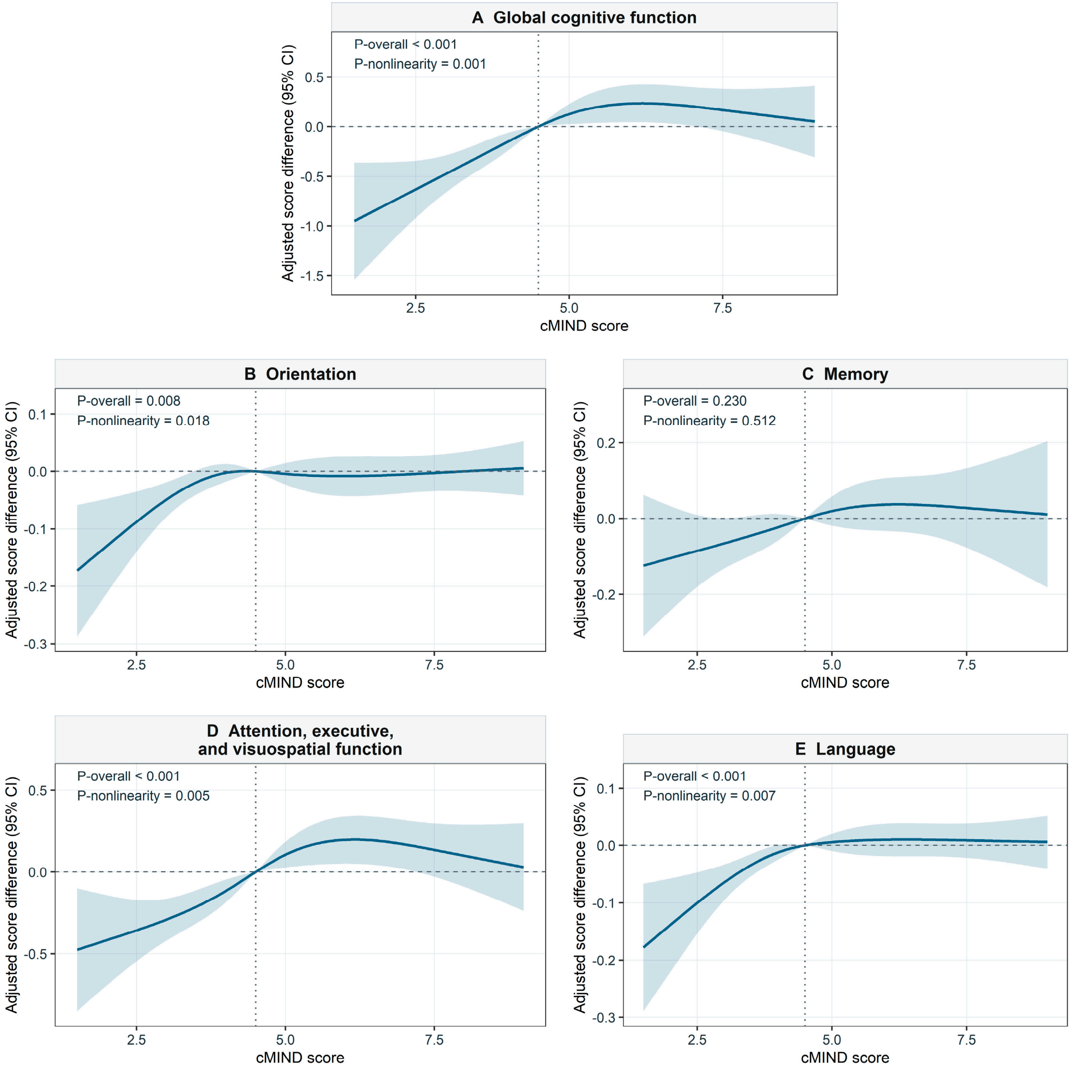

Figure S2. Dose–response associations of cMIND score with cognitive function after excluding participants with age-specific very low MMSE scores or reported dementia

Restricted cubic spline associations of cMIND score with global cognitive function (A), orientation (B), memory (C), attention, executive, and visuospatial function (D), and language (E). Solid lines indicate adjusted estimates and shaded areas indicate 95% CIs. The vertical dotted line indicates the weighted median reference. Models used the same covariate adjustment as Model 5.

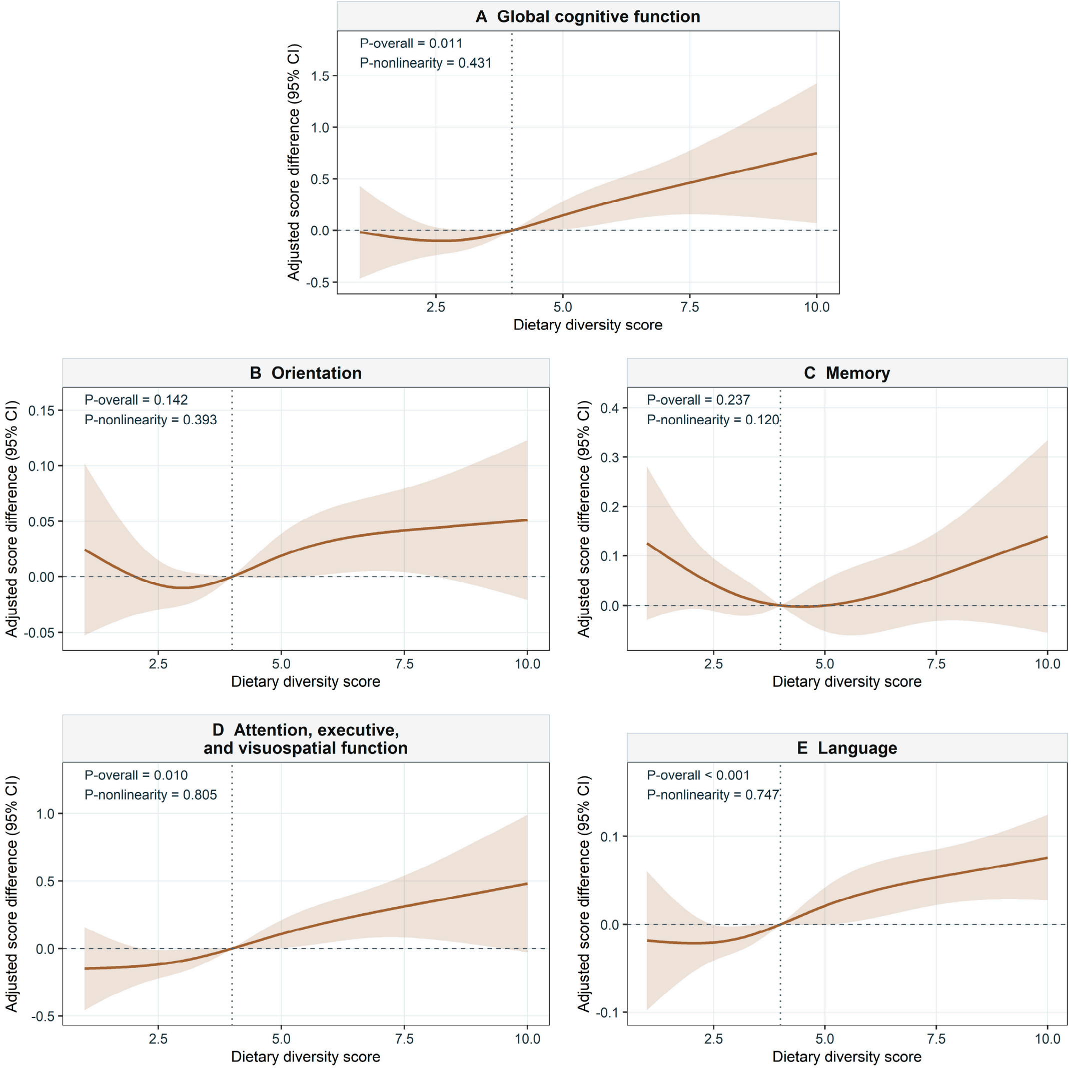

Figure S3. Dose–response associations of dietary diversity with cognitive function after excluding participants with age-specific very low MMSE scores or reported dementia

Restricted cubic spline associations of dietary diversity score with global cognitive function (A), orientation (B), memory (C), attention, executive, and visuospatial function (D), and language (E). Solid lines indicate adjusted estimates and shaded areas indicate 95% CIs. The vertical dotted line indicates the weighted median reference. Models used the same covariate adjustment as Model 5.

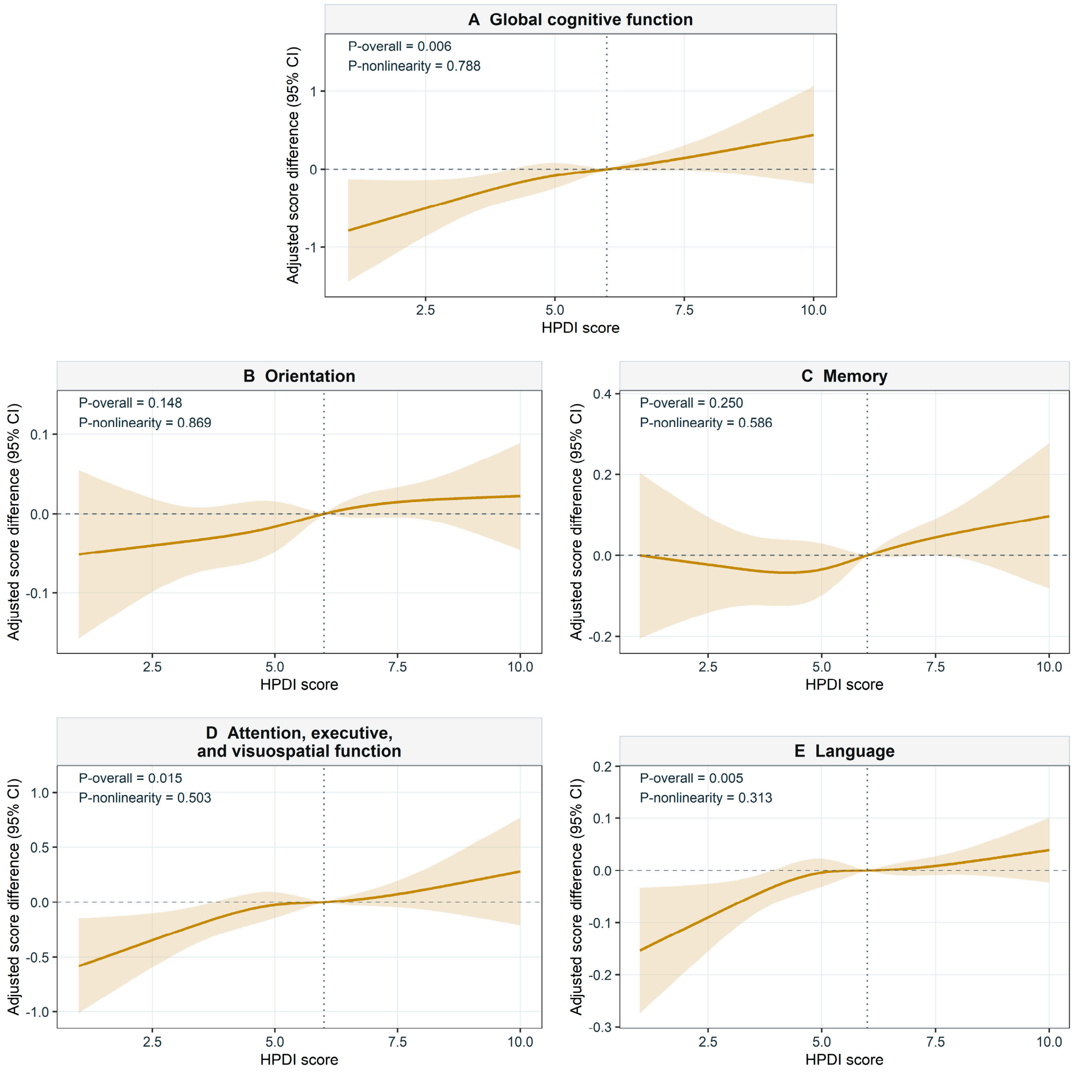

Figure S4. Dose–response associations of HPDI with cognitive function after excluding participants with age-specific very low MMSE scores or reported dementia

Restricted cubic spline associations of HPDI with global cognitive function (A), orientation (B), memory (C), attention, executive, and visuospatial function (D), and language (E). Solid lines indicate adjusted estimates and shaded areas indicate 95% CIs. The vertical dotted line indicates the weighted median reference. Models used the same covariate adjustment as Model 5.

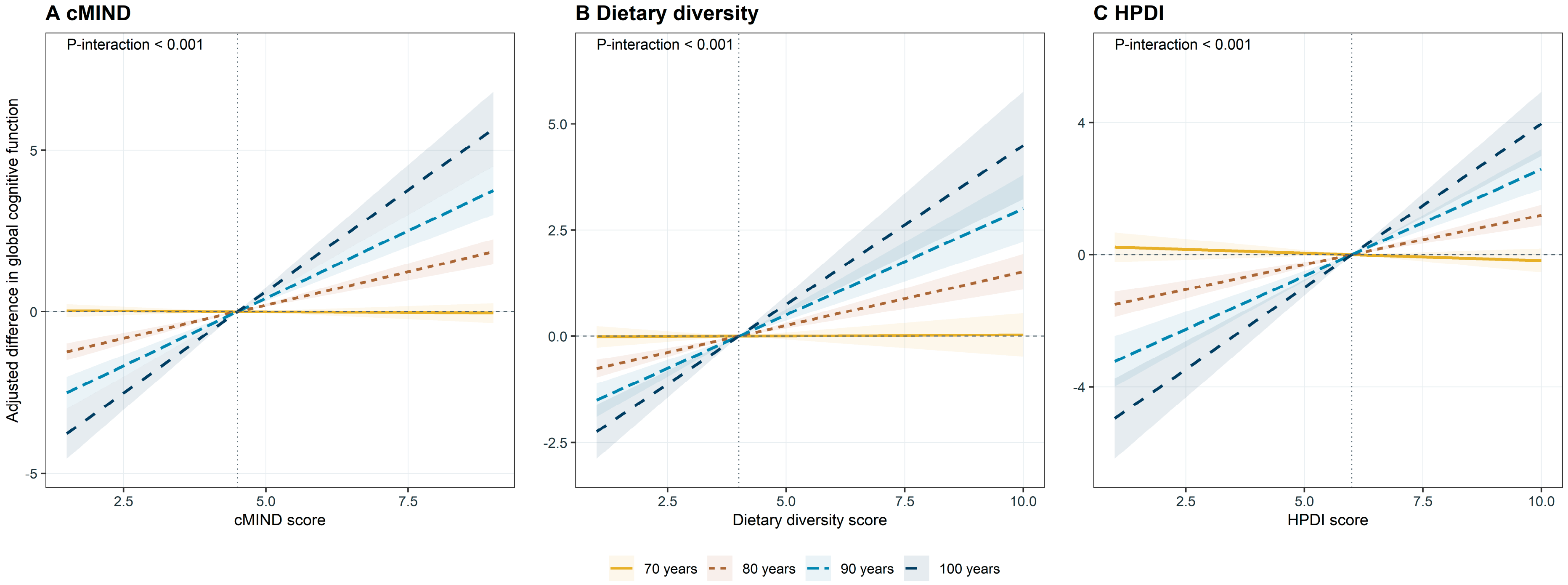

Figure S5. Age-related heterogeneity in associations between dietary indices and global cognitive function after excluding participants with age-specific very low MMSE scores or reported dementia

Adjusted associations of cMIND (A), dietary diversity (B), and HPDI (C) with global cognitive function at ages 70, 80, 90, and 100 years. Lines indicate estimated associations and shaded areas indicate 95% CIs. Models used continuous age and diet-by-age interaction terms with the remaining Model 5 covariates. P values are for diet-by-age interaction.
